# Clusterin overexpression protects against western diet-induced obesity and NAFLD

**DOI:** 10.1038/s41598-020-73927-y

**Published:** 2020-10-15

**Authors:** Jin-Sung Park, Woon-Kyu Lee, Hyeon Soo Kim, Ji A. Seo, Dong-Hoon Kim, Hee Chul Han, Bon-Hong Min

**Affiliations:** 1 Department of Physiology, College of Medicine, Korea University, Seoul, Korea; 2 Department of Biomedical Sciences, College of Medicine, Inha University, Incheon, Korea; 3 Department of Anatomy, College of Medicine, Korea University, Seoul, Korea; 4 Department of Endocrinology and Metabolism, Korea University, Ansan, Korea; 5 Department of Pharmacology, College of Medicine, Korea University, Seoul, Korea

**Keywords:** Biochemistry, Cell biology, Immunology, Molecular biology, Biomarkers, Diseases, Endocrinology

## Abstract

Obesity is a significant risk factor for various metabolic diseases and is closely related to non-alcoholic fatty liver disease (NAFLD) characterized by inflammation and oxidative stress. Clusterin is a multi-functional protein that is up-regulated in the pathogenesis of various metabolic diseases, including obesity and NAFLD. Our previous studies indicated that hepatocyte-specific overexpression of clusterin alleviates methionine choline-deficient (MCD) diet-induced non-alcoholic steatohepatitis (NASH) by activating nuclear factor erythroid 2-related factor 2 (Nrf2). Here we generated transgenic mice with whole-body clusterin overexpression (wCLU-tg) and investigated the role of clusterin in Western diet-induced obesity and NAFLD. We confirmed that obesity parameters and the spectrum of NAFLD of wCLU-tg mice were improved compared to wild type mice. Contrarily, clusterin deficiency deteriorated metabolic disruptions. We also found that clusterin activates target molecules for obesity and NAFLD, namely Nrf2 and AMPK, suggesting that clusterin protects against Western diet-induced obesity and NAFLD by activating Nrf2 and AMPK.

## Introduction

The prevalence of obesity is rapidly increasing worldwide, partially driven by Western dietary patterns and sedentary lifestyle habits. Obesity induces chronic low-grade inflammation that can upregulate pro-inflammatory cytokines to affect a variety of tissues, making them vulnerable to many diseases. Obesity is of great concern because its complications are often more serious than obesity itself. Indeed, obesity is associated with an increased risk for metabolic diseases, such as non-alcoholic fatty liver disease (NAFLD), insulin resistance (IR), and cardiovascular disease (CVD)^1–3^.

NAFLD is the most common chronic liver disease across the globe^4^ and its pathology ranges from simple steatosis to nonalcoholic steatohepatitis (NASH) or hepatic fibrosis. Simple steatosis is characterized by the accumulation of excess fat in the liver while NASH is characterized by oxidative stress and inflammation that can progress to more severe NAFLD pathologies such as fibrosis and cirrhosis^5,6^. NAFLD is also associated with an increased prevalence of metabolic diseases including obesity, IR, CVD, and type 2 diabetes (T2D)^7,8^. Several studies have shown a correlation between obesity and NAFLD. Weight loss and dietary restriction have been demonstrated to reduce fat accumulation in the liver^9,10^. Obesity and NAFLD are major risk factors for metabolic syndrome, and many reports suggest that patients with metabolic syndrome are at increased risk of developing CVD or T2D^11,12^. Therefore, it is important to understand and treat the pathology of obesity and NAFLD to prevent metabolic syndrome. Treatments for obesity and NAFLD commonly include lifestyle management through weight loss, dietary restriction, exercise, etc.^13,14^. Although there has been extensive research on this topic, there are currently few drugs approved for the treatment of obesity and NAFLD.

Treatment of obesity and NAFLD has been shown to be associated with AMP-activated protein kinase (AMPK) activation^15,16^. AMPK is a heterotrimeric serine/threonine kinase complex that acts as a key regulator of energy balance at the cellular and organismal levels. AMPK plays an important role in several metabolic pathways, including lipid metabolism, glucose homeostasis, and inflammation. Specifically, AMPK activation inhibits fatty acid synthesis and lipolysis in adipose tissue, suppresses fatty acid synthesis and gluconeogenesis in the liver^17^, and suppresses the inflammatory responses of various inflammatory diseases^18,19^. Together, these features have made AMPK an attractive therapeutic target for treating obesity, IR, NAFLD, and CVD^20^.

Clusterin is a heterodimeric glycoprotein expressed in various mammalian tissues and is present in most biological fluids, including plasma, urine, and milk, at low levels under normal physiological conditions. However, clusterin levels are upregulated in pathophysiological conditions, such as oxidative stress and inflammation^21,22^. In particular, many studies have shown that clusterin is associated with various metabolic diseases. For example, the plasma concentration of clusterin is closely related to obesity and T2D^23,24^, and clusterin levels are elevated in patients with metabolic syndrome^25^. Additionally, clusterin reduces hepatic lipogenesis by downregulating sterol regulatory binding protein-1c (SREBP-1C), a master regulator of lipogenesis^26^. Clusterin deficiency also exacerbates high-fat diet (HFD)-induced insulin resistance (HFD)^27^. Our previous studies showed that hepatocyte-specific overexpression of clusterin attenuates diet-induced NASH by activating nuclear factor erythroid 2-related factor 2 (Nrf2), a master regulator of anti-oxidant and anti-inflammation pathways^28^. In summary, clusterin plays a protective role in a variety of metabolic diseases, but relatively little is known about the protective mechanisms of clusterin in these diseases, necessitating further investigation.

In this study, we found that clusterin levels were significantly increased in the serum and metabolic tissues, such as adipose and liver tissue, of wild type mice fed a high fat and sucrose diet (Western diet, WD) as compared to wild type mice fed a chow diet (CHOW). To investigate the role of increased clusterin in diet-induced obesity and NAFLD, we generated transgenic mice with whole-body clusterin overexpression (wCLU-tg; C56BL/6J-CLU^floxed+/+^ × C56BL/6J^Ella cre+^) and confirmed that clusterin upregulation has a protective effect. Specifically, wCLU-tg mice had substantial protection against diet-induced increase in body weight and body mass index (BMI), hepatic steatosis, and inflammation. To determine whether clusterin itself has a protective effect or instead functions by modulating other protective mechanisms, we examined the protective molecular mechanisms of clusterin. We confirmed that clusterin achieves a protective effect by regulating the expression of AMPK and Nrf2. Taken together, our findings suggest that clusterin plays an important role in the prevention and treatment of diet-induced obesity and NAFLD, two main risk factors for metabolic syndrome.

## Results

### Clusterin is upregulated in diet-induced obesity and NAFLD

**Figure 1:**
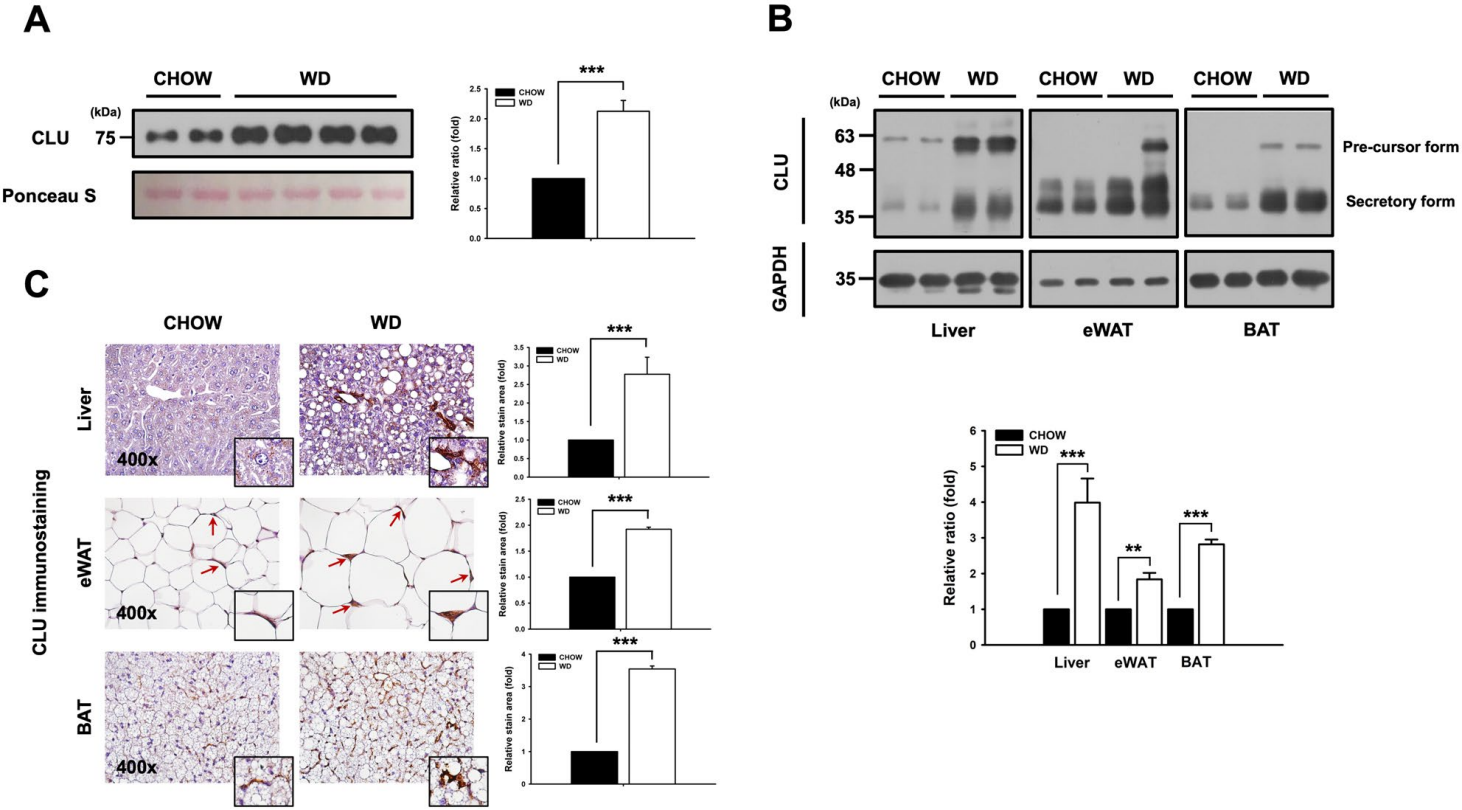
Clusterin is upregulated in metabolic diseases induced by WD. (**A**) Serum clusterin expression level in wild type mice with metabolic diseases induced by WD. (**B**) Western blot analysis of clusterin expression in various metabolic tissues, including Liver, eWAT, BAT of wild type mice fed CHOW or WD. (**C**) Immunostaining for clusterin in various tissues of wild type mice fed CHOW or WD. Clusterin expression level was approximately 2.5–3 times higher in the liver, 2 times higher in the eWAT, and 3–4 times higher in the BAT of WD-fed wild type mice than in Chow-fed wild type mice. Data are expressed as standard error (± SEM), and eight mice were used per group. Three independent experiments were performed. **P < 0.01; ***P < 0.001 vs CHOW -fed WT mice. Magnification, 400x.

High fat and high sucrose WD can be used as dietary model to cause obesity and NAFLD^29–31^. To identify the relationship between the clusterin expression levels and diet-induced metabolic changes, we provided mice with CHOW or WD ad libitum for 15 weeks. Mice receiving WD were characterized by weight gain, increase of fat mass, and hepatic steatosis with appreciable hepatic inflammation and fibrosis, similar to previous results^32,33^. Western blotting and immunohistochemistry were used to assess clusterin expression levels in blood and metabolic tissues. Serum clusterin levels in wild type mice fed WD were twofold higher than in wild type mice fed CHOW (Fig. 1A). Also, clusterin expression was two to four times higher in wild type mice fed WD than in wild type mice fed CHOW (Fig. 1B). Subsequently, immunohistochemical staining of clusterin was performed on adipose tissues (eWAT: epididymal white adipose tissue, BAT: brown adipose tissue) and liver tissue. Similar to Fig. 1B, clusterin expression levels in the metabolic tissues of wild type mice fed WD were significantly increased compared to wild type mice fed CHOW (Fig. 1C). These data suggest that clusterin may play an important role in obesity and NAFLD.

### Generation of wCLU-tg mice

**Figure 2:**
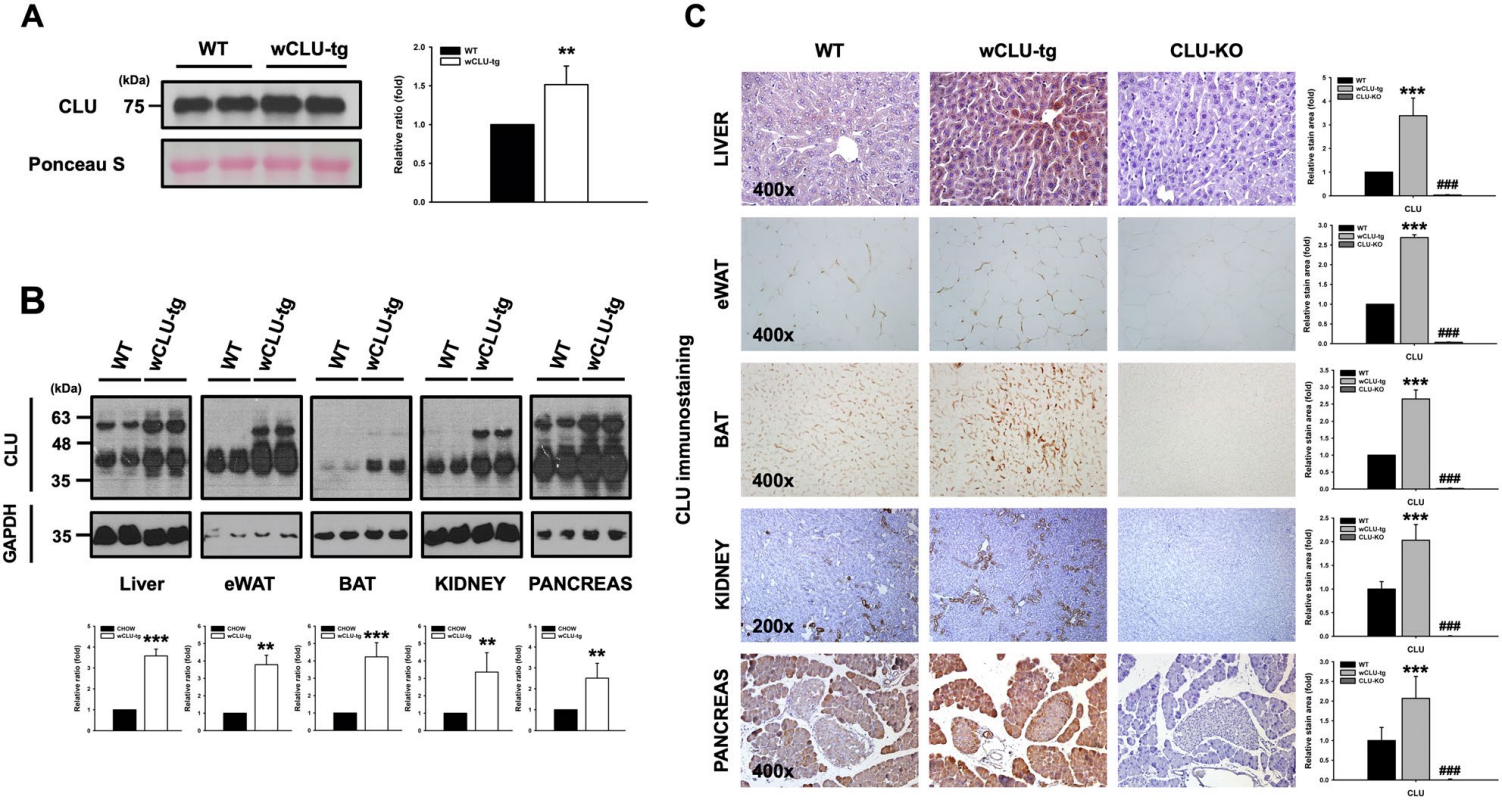
Clusterin overexpression in various tissues of wCLU-tg mice. (**A**) Serum clusterin expression level in wild type mice and wCLU-tg mice fed CHOW. (**B**) western blot analysis and (**C**) Immunostaining for clusterin in various tissues of wild type mice and wCLU-tg mice fed CHOW. Clusterin expression level was approximately 3–4 times higher in the liver and 2.5–3 times higher in the both eWAT and BAT of CHOW-fed wCLU-tg mice than in Chow-fed wild type mice. The liver tissue, eWAT, and BAT of CLU-KO mice were used as negative controls. **P < 0.01; ***P < 0.001; ^###^*P* < 0.001 vs CHOW -fed WT mice. Magnification, 200x, 400x.

To investigate the role of increased clusterin in WD-induced obesity and NAFLD, we generated transgenic mice with whole-body clusterin overexpression (wCLU-tg). wCLU-tg mice were obtained by crossing floxed mice carrying a clusterin knock-in allele (C56BL/6J-CLU^floxed+/+^) with transgenic mice carrying Cre recombinase under the control of the adenovirus EIIa promoter to drive Cre recombinase expression in the early mouse embryo (Jackson Laboratory, stock number 003724). We have previously described the generation and screening of C56BL/6 J-CLU^floxed+/+^ mice ^28^. Whole-body overexpression of clusterin was analyzed by western blotting and immunohistochemistry. First, we found that serum clusterin levels in wCLU-tg mice were approximately 1.5–2 times higher than in wild type mice (Fig. 2A). Next, we identified clusterin overexpression in various tissues, including liver, eWAT, BAT, kidney, and pancreas. Clusterin expression levels were higher in various tissues of wCLU-tg mice than in those of wild type mice, while GAPDH, serving as an internal control, did not vary significantly (Fig. 2B). Additionally, clusterin immunostaining was increased by 3–fourfold in the liver, 2.5–threefold in the eWAT and BAT, and 2–2.5-fold in the kidney and pancreas in wCLU-tg mice as compared to wild-type mice (Fig. 2C). As a negative control, the tissues of clusterin knock-out (CLU-KO) mice were used, and it was confirmed that the clusterin was not stained in the clusterin knock-out tissues.

### Role of clusterin in diet induced-weight gain and obesity

**Figure 3:**
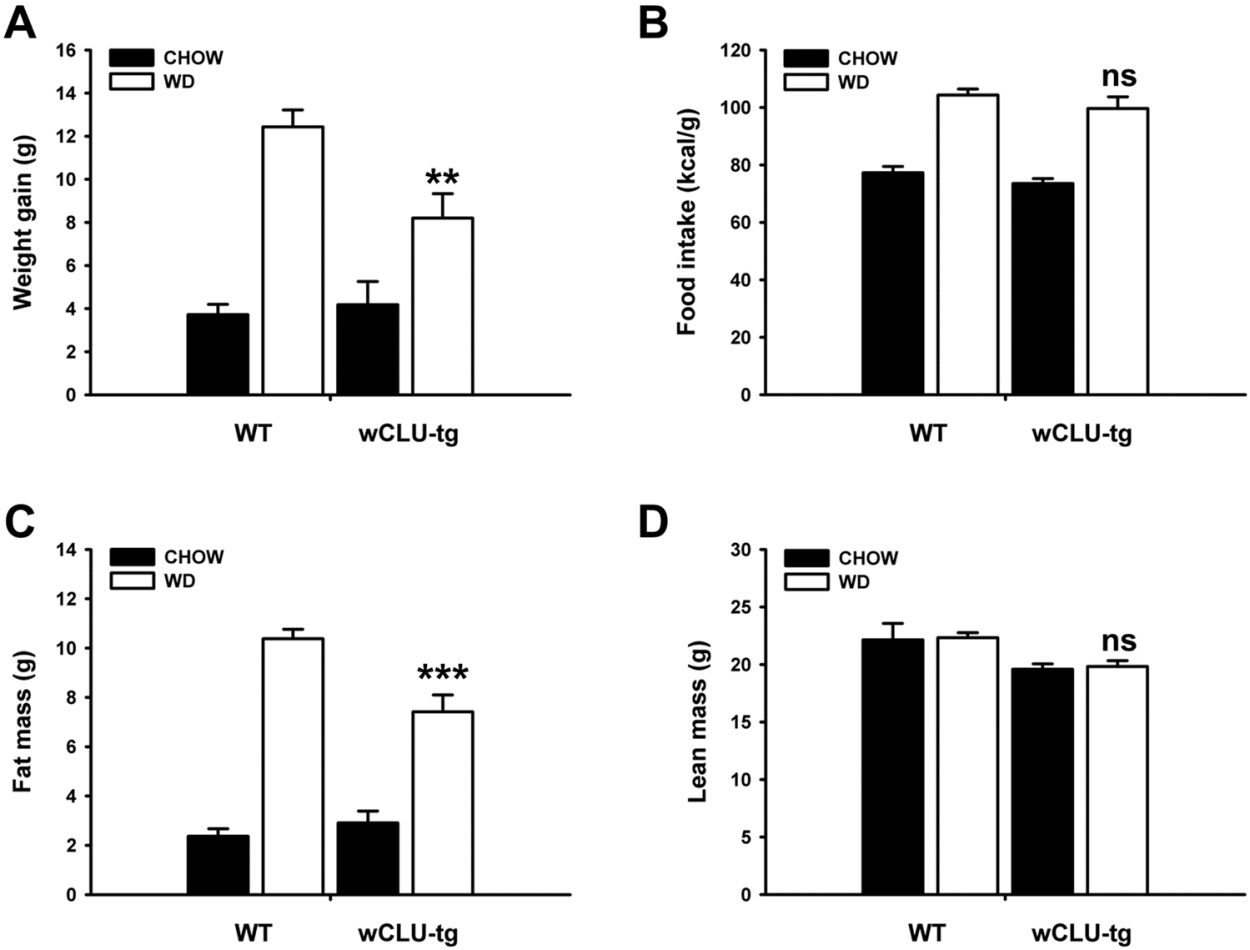

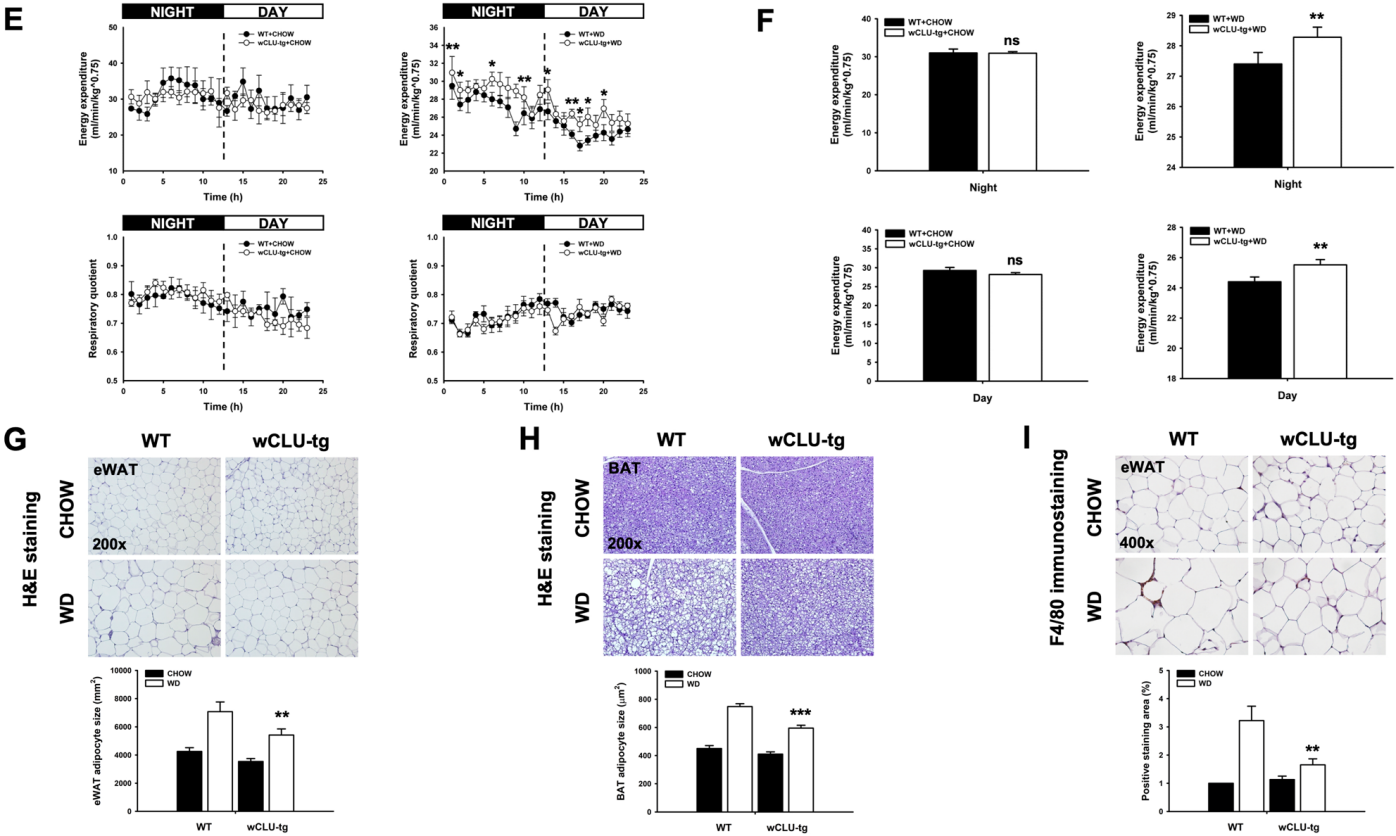
Role of clusterin in weight gain and related parameters induced by WD. (**A**,**B**) Weight gain and calorie intake in the two groups of mice fed CHOW or WD for 15 weeks. Body weight of WD-fed wCLU-tg mice was less than that of WD-fed wild type mice despite no difference in calorie intake. (**C**,**D**) Analysis of fat mass and lean mass using a fat/lean analyzer. Fat mass of WD-fed wCLU-tg mice was lower than that of WD-fed wild type mice while there was no significant difference in lean mass. (**E**,**F**) Oxygen consumption (VO_2_), CO_2_ emission (VCO_2_), and RQ (respiratory quotient) values were measured using an indirect calorimeter for 3 h at intervals of 30 min every 24 h. (**G**,**H**) H&E (Hematoxylin & Eosin) staining for eWAT and BAT of the two groups mice fed CHOW and WD. (I) F4/80 staining in eWAT. *P < 0.05; **P < 0.01; ***P < 0.001 vs WD-fed WT mice. ns: not significant. Magnification, × 200, × 400.

Wild type mice fed WD had a significantly greater weight gain than that of wild type mice fed CHOW for the same period of time (15 weeks, 12 g vs. 4 g), similar to previous results^29^. While the weight gain of wCLU-tg mice fed WD was also significantly increased compared to that of wCLU-tg mice fed CHOW (8 g vs. 4 g), the difference in weight gain in wCLU-tg mice was significantly smaller than that in wild type mice (Fig. 3A). Meanwhile, clusterin knockout (CLU-KO) mice fed WD had a greater weight gain than that of wild type mice fed WD (Supplementary Fig. S1A). Interestingly, no significant difference in food intake was found between the two groups of WD-fed mice (Fig. 3B); however, food consumption was higher in WD-fed mice than those fed CHOW. Notably, clusterin deficiency increased food intake (Supplementary Fig. S1B).

Next, we measured body fat and lean mass using a body composition analyzer. The body fat mass of WD-fed wCLU-tg mice was lower than WD-fed wild type mice (Fig. 3C), but the body fat mass of WD-fed CLU-KO mice was significantly increased compared to WD-fed wild type mice (Supplementary Fig. S1C). There was no significant difference in lean mass between the WD-fed wCLU-tg and WD-fed wild type mice (Fig. 3D), and this pattern is also shown in Supplementary Fig. S1D. High-calorie diet food intake reduces energy expenditure^34^. We found no difference in energy expenditure between wild type and wCLU-tg mice fed a CHOW diet; however, the energy expenditure of WD-fed wCLU-tg mice was greater than that of WD-fed wild type mice (Fig. 3E,F). The energy expenditure of WD-fed CLU-KO mice was significantly lower than that of WD-fed wild type mice (Supplementary Fig. S1E).

Based on these results, we performed Hematoxylin & Eosin staining on eWAT and BAT. The white and brown fat sizes of WD-fed wCLU-tg mice were reduced compared to those of WD-fed wild type mice, but there was no difference in the white and brown fat sizes of the two groups of CHOW-fed mice (Fig. 3G,H). Clusterin deficiency resulted in eWAT and BAT hypertrophy (Supplementary Fig. S1F,G). In addition, when energy balance is disrupted by WD, the inflammatory cell population increases and forms a crown-like structure in adipose tissue^35^. To investigate inflammatory cells, we performed immunohistochemical staining for F4/80, a well-known and widely used inflammatory cell marker. While there were no significant differences in the F4/80 positive macrophage populations between CHOW-fed wild type and wCLU-tg mice, inflammatory cell populations were reduced in the eWAT of WD-fed wCLU-tg mice compared to WD-fed wild type mice (Fig. 3I).

### Clusterin has protective effects against WD-induced NAFLD

**Figure 4:**
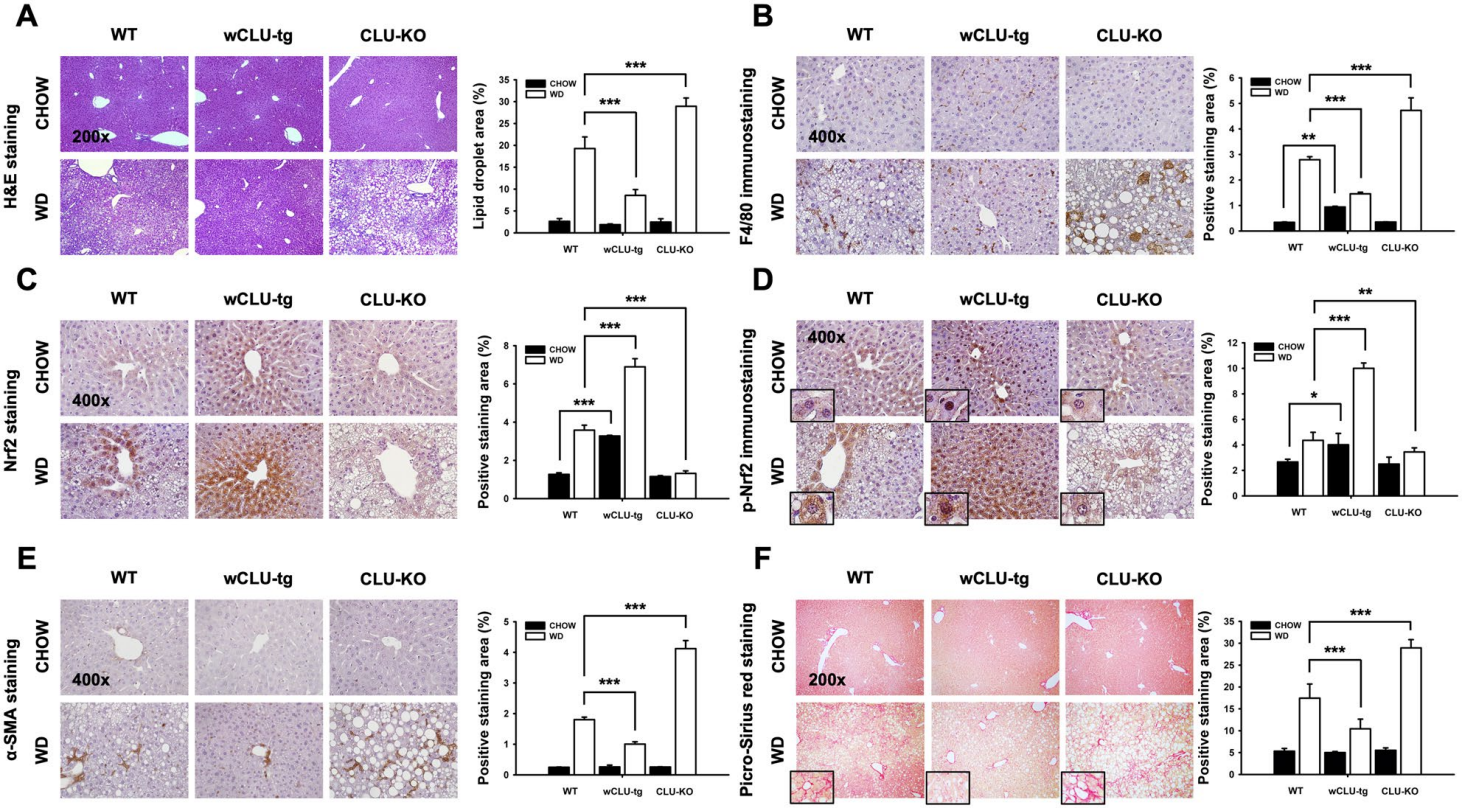
Protective effect of clusterin in the pathology of WD-induced NAFLD. (**A**) H&E staining in the liver of wild type, wCLU-tg, and CLU-KO mice fed CHOW or WD. (**B**–**E**) Immunostaining for F4/80, Nrf2, p-Nrf2, and α-SMA in the liver of wild type, wCLU-tg, and CLU-KO mice fed CHOW or WD. (**F**) Picro-Sirius red staining in the liver of wild type, wCLU-tg, and CLU-KO mice fed CHOW or WD. *P < 0.05; **P < 0.01; ***P < 0.001. Magnification, × 200, × 400.

We found significantly less hepatic lipid accumulation in WD-fed wCLU-tg mice than in WD-fed wild type mice, but clusterin deficiency resulted in deterioration of liver damage (Fig. 4A). We next performed immunohistochemical staining for F4/80 to assess the degree of inflammation caused by WD. As shown in Fig. 4b, F4/80 immunostaining was significantly increased in the liver of CHOW-fed wCLU-tg mice compared to both wild type and CLU-KO mice fed CHOW, suggesting that clusterin may induce mild inflammation. In three groups of mice (wild type, wCLU-tg, CLU-KO) fed WD, F4/80 immunostaining showed a significant increase compared to CHOW-fed mice; however, its level was significantly lower in WD-fed wCLU-tg mice than in WD-fed wild type mice. Interestingly, the immune-reactivity for F4/80 in WD-fed CLU-KO mice was greater than that of both wild type and wCLU-tg mice fed WD (Fig. 4B). Based on these results, we confirmed expression of Nrf2, a major regulator of the endogenous antioxidant defense mechanism that plays a role in inhibiting hepatic lipid metabolism^36^ and inflammation^37,38^. Nrf2 expression was significantly higher in both wild type and wCLU-tg mice fed WD than in wild type and wCLU-tg mice fed CHOW. However, Nrf2 expression in WD-fed CLU-KO mice was not significantly different from that of CHOW-fed CLU-KO mice. Also, phosphorylated Nrf2, as an activated form of Nrf2, was increased in WD-fed wCLU-tg mice compared to CHOW-fed wild type and CLU-KO mice (Fig. 4C,D). Interestingly, the basal expression level of Nrf2 and phospho-Nrf2 in CHOW-fed wCLU-tg mice was greater than that of CHOW-fed wild type mice. Additionally, hepatic fibrosis occurs through the pathologic deterioration of nonalcoholic steatohepatitis and activates alpha-smooth muscle actin (α-SMA) cells known to mark fibrotic liver. Fibrotic changes in the liver were assessed by α-SMA immunostaining and Picro-Sirius red staining. α-SMA cell activation was lower in WD-fed wCLU-tg mice than in WD-fed wild type mice whereas WD-fed CLU-KO mice had significantly more α-SMA cell activation than both wild type and wCLU-tg mice fed WD (Fig. 4E). The fibrosis area in WD-fed wCLU-tg mice was significantly smaller than those in WD-fed wild type and CLU-KO mice. Moreover, the fibrotic changes in WD-fed CLU-KO mice were severe than in WD-fed wild type mice (Fig. 4F).

### Clusterin expression correlates with AMPK activation

**Figure 5:**
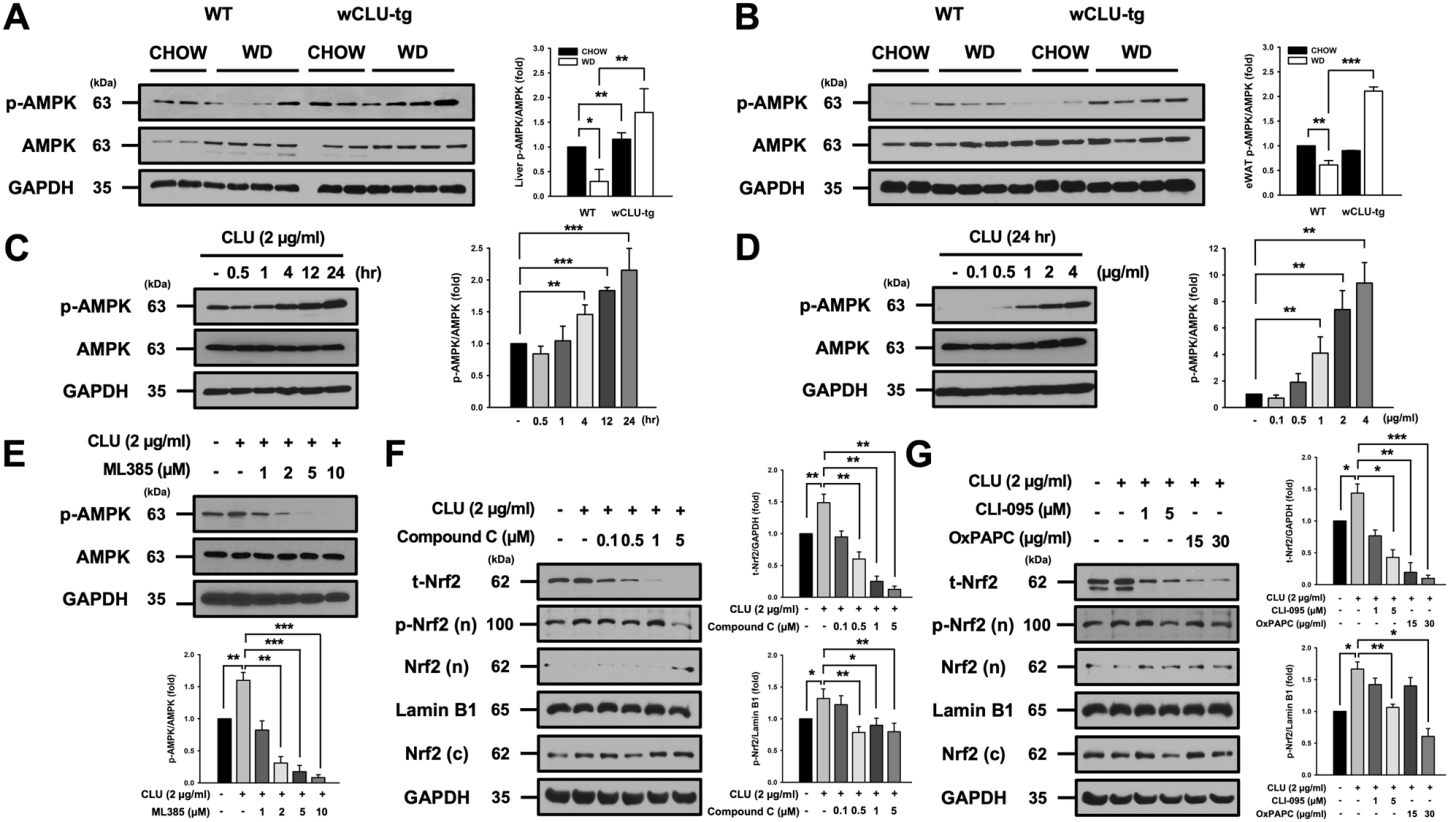
Correlation between clusterin expression and AMPK activation. (**A**,**B**) Western blotting analysis of AMPK phosphorylation in the liver and eWAT of wild type and wCLU-tg mice fed CHOW or WD for 15 weeks. GAPDH was used as an internal control and was used to determine the relative ratio of AMPK phosphorylation. (**C**,**D**) Huh7 cells were incubated in 6-well plates. After 24 h, cells were incubated in serum-free DMEM. AMPK phosphorylation increased in a time- (0, 0.5, 1, 4, 12, 24 h) and dose- (0, 0.1, 0.5, 1, 2, 4 µg/ml) dependent manner in Huh7 cells. (**E**) Huh7 cells were pretreated with ML385 at 1, 2, 5, and 10 µM concentrations for 1 h and then treated with clusterin (2 µg/ml) for 48 h. (**F**) Huh7 cells were pretreated with Compound C at concentrations of 1, 2, 5, and 10 µM for 1 h and then treated with clusterin (2 µg/ml) for 48 h. (**G**) Huh7 cells were pretreated with TLR4 inhibitors, CLI-095 (1, 5 µM), and OxPAPC (15, 30 µg/ml), for 30 min and then treated with clusterin (2 µg/ml) for 24 h. *P < 0.05; **P < 0.01; ***P < 0.001.

To identify whether the protective effect of clusterin in diet-induced obesity and NAFLD is associated with AMPK activation, we examined AMPK activation in the liver and eWAT. AMPK activation was higher in the liver and eWAT of WD-fed wCLU-tg mice than in WD-fed wild type mice (Fig. 5A,B). Interestingly, AMPK activation was slightly increased in the liver of CHOW-fed wCLU-tg mice compared to CHOW-fed wild type mice but was not significantly different in the eWAT. Based on these results, to investigate whether clusterin activates AMPK in vitro, we treated Huh7 cells, a liver cancer cell line, with purified human clusterin. We found that clusterin increases AMPK activation in a time- and dose-dependent manner (Fig. 5C,D). Many studies have shown that the interaction between Nrf2 and AMPK is closely related to the protective effect of preconditioning (PC)^39^. Through our previous work^28^ and this study, we confirmed that clusterin increases Nrf2 and AMPK levels, both of which play an important role in preconditioning. To identify the interaction between Nrf2 and AMPK, Huh7 cells were pretreated with Nrf2 inhibitor (ML385) and AMPK inhibitor (Compound C), then treated with purified human clusterin (2 µg/ml). We found that pharmacological inhibition of Nrf2 blunts clusterin-induced AMPK phosphorylation, but there was no change in the expression level of total AMPK (Fig. 5E). Also, inhibition of AMPK did not affect the nuclear translocation of Nrf2, but reduced clusterin-induced the expression of total Nrf2 and phosphorylated Nrf2 (Fig. 5F). There are correlations between the TLR4 signaling pathway and Nrf2 and AMPK activation, but their relationship is not yet fully understood. To determine if the TLR4 signaling pathway correlates with clusterin-induced Nrf2 and AMPK activation, we pretreated Huh7 cells with different concentrations of TLR4 inhibitors (CLI-095: inhibition of intracellular domain, OxPAPC: inhibition of extracellular domain), then treated the cells with purified human clusterin (2 µg/ml). We found that TLR4 receptor and signal transduction inhibition reduces clusterin-induced the expression of total Nrf2 and phosphorylated Nrf2 (Fig. 5G), and the AMPK phosphorylation (Supplementary Fig. S3). In summary, these results indicate that clusterin alleviates inflammation and oxidative stress via TLR4 and AMPK/Nrf2 activation.

## Discussion

The present study demonstrated that clusterin protects against the pathophysiology of obesity and NAFLD by reducing body weight and fat mass, hepatic fat accumulation, and steatohepatitis. Many studies have shown the correlation between clusterin expression and metabolic diseases such as obesity and NAFLD. Clusterin expression was higher in obese patients without other metabolic disturbances than in healthy control subject^24^. Additionally, plasma clusterin levels were significantly reduced in obese patients after weight reduction^23^. Furthermore, plasma clusterin levels were significantly increased in the pathophysiology of HFD-induced NAFLD^27^, whereas no significant difference was found in hepatic clusterin expression between CHOW-fed wild type mice and wild type mice fed a high-fat diet^26^. In summary, the role of clusterin expression levels on the pathophysiology of obesity and NAFLD are still uncertain, but there is a considerable correlation between clusterin and these diseases.

In the present study, we found that clusterin expression was increased in serum and/or metabolic tissues of WD-fed mice, and that the upregulation of clusterin is closely related to a protective effect. These results support the controversy of clusterin expression in obesity and NAFLD. However, further studies on the expression and function of clusterin in the pathophysiology of these diseases are needed. Subsequently, we found that serum clusterin levels were increased in CHOW-fed wCLU-tg mice compared to CHOW-fed wild type mice. In addition, clusterin expression was significantly increased in the liver relative to other tissues, including eWAT, BAT, kidney, and pancreas. Clusterin is expressed in various tissues, but its origin is still unclear. Some studies have shown that clusterin mRNA expression was relatively increased in hepatocytes compared to other tissues and that liver tissues are the main source of circulating clusterin in the blood^40^. These findings are consistent with our results and provide evidence that the liver may be the main source of clusterin.

Next, the protective effect of clusterin in WD-induced obesity and NAFLD can be explained by our results and the preconditioning (PC) effect mentioned previously^28^. First, clusterin deficiency exacerbates WD-induced metabolic parameters in obesity and NAFLD whereas clusterin overexpression results in protective effects. Second, preconditioning is a phenomenon in which sub-lethal stress confers a protective effect against subsequent severe injury. Damage-associated molecular patterns (DAMPs) are known as endogenous danger molecules that interact with TLR4 receptors and induce various sub-lethal stresses, such as inflammation and oxidative stress, which can serve to precondition. Although the precise mechanisms underlying preconditioning are unknown, several studies have provided important clues to the protective effect of preconditioning. The inflammatory response can be divided into pro- and anti-inflammatory responses; the pro-inflammatory response is accompanied by the NF-κB pathway to promote the production of ROS and pro-inflammatory cytokines while the anti-inflammatory response is associated with the Nrf2/ARE pathway to promote antioxidant enzyme production. Specifically, a low dose of LPS induces a mild inflammatory response that promotes Nrf2 activation and protects cells from subsequent severe liver injury^41^. Additionally, TLR4 receptor activation prior to the induction of oxidative stress in retinal cells reduces photoreceptor cell death^42^. These results suggest that the TLR4 receptor plays an important role in preconditioning, and the common protective mechanisms of preconditioning include the reduction of an inflammatory response and oxidative stress through Nrf2 activation. In previous studies, we confirmed that clusterin-induced mild inflammation protects against diet-induced NAFLD by enhancing Nrf2 expression at the tissue level. In this study, we also confirmed Nrf2 upregulation by clusterin at the cellular level (Supplementary Figure S2). Based on these data, this study revealed protective mechanisms that suppress inflammation under preconditioning by clusterin.

Further, AMPK activation attenuates inflammation and oxidative stress in a variety of metabolic diseases^43,44^. Specifically, AMPK activation mitigates pro-inflammatory cytokines by downregulating the PI3K/p38/NF-κB signaling pathway^45^ by indirectly targeting NF-κB^43^. Thus, other pathways beyond NF-κB are speculated to be involved, and some studies have reported that Nrf2 interacts with AMPK in the anti-inflammatory effect of preconditioning^39^. Additionally, clusterin alleviates endothelial dysfunction in Leptin receptor-deficient diabetic (db/db) and Streptozotocin (STZ)-induced diabetes by activating AMPK^46^. Therefore, these results indicate that, in inflammation and oxidative stress, AMPK activation is closely related to Nrf2 and NF-κB signaling, and clusterin plays an important role in this relationship. We found that clusterin interacts with AMPK and Nrf2. As shown in Fig. 5A,B, AMPK activation was slightly increased in the liver of CHOW-fed wCLU-tg mice compared to CHOW-fed wild-type mice but was not significantly different in the eWAT. This phenomenon is presumably due to the fact that clusterin expression levels are different in each tissue, and clusterin overexpression was higher in the liver of wCLU-tg mice than in other tissues. Thus, these results indicate that AMPK activation is regulated by clusterin expression. To confirm the correlation between clusterin and AMPK activation, we treated Huh7 cells with purified human clusterin at different time points and concentrations. Clusterin enhanced AMPK activation in a time- and dose-dependent manner (Fig. 5C,D). These results not only support our in vivo experiments but also show that clusterin may facilitate preconditioning. Several studies have shown that AMPK activation enhances Nrf2/HO-1 signaling^47,48^. So, we examined whether clusterin regulates AMPK/Nrf2 crosstalk. Interestingly, we found that Nrf2 inhibition diminishes clusterin-induced AMPK phosphorylation and AMPK inhibition blunts the expression of total Nrf2 and phosphorylated Nrf2 (Fig. 5E,F). In addition, TLR4 inhibition reduced clusterin-induced phosphorylation of AMPK and Nrf2 (Fig. 5G).

**Figure 6:**
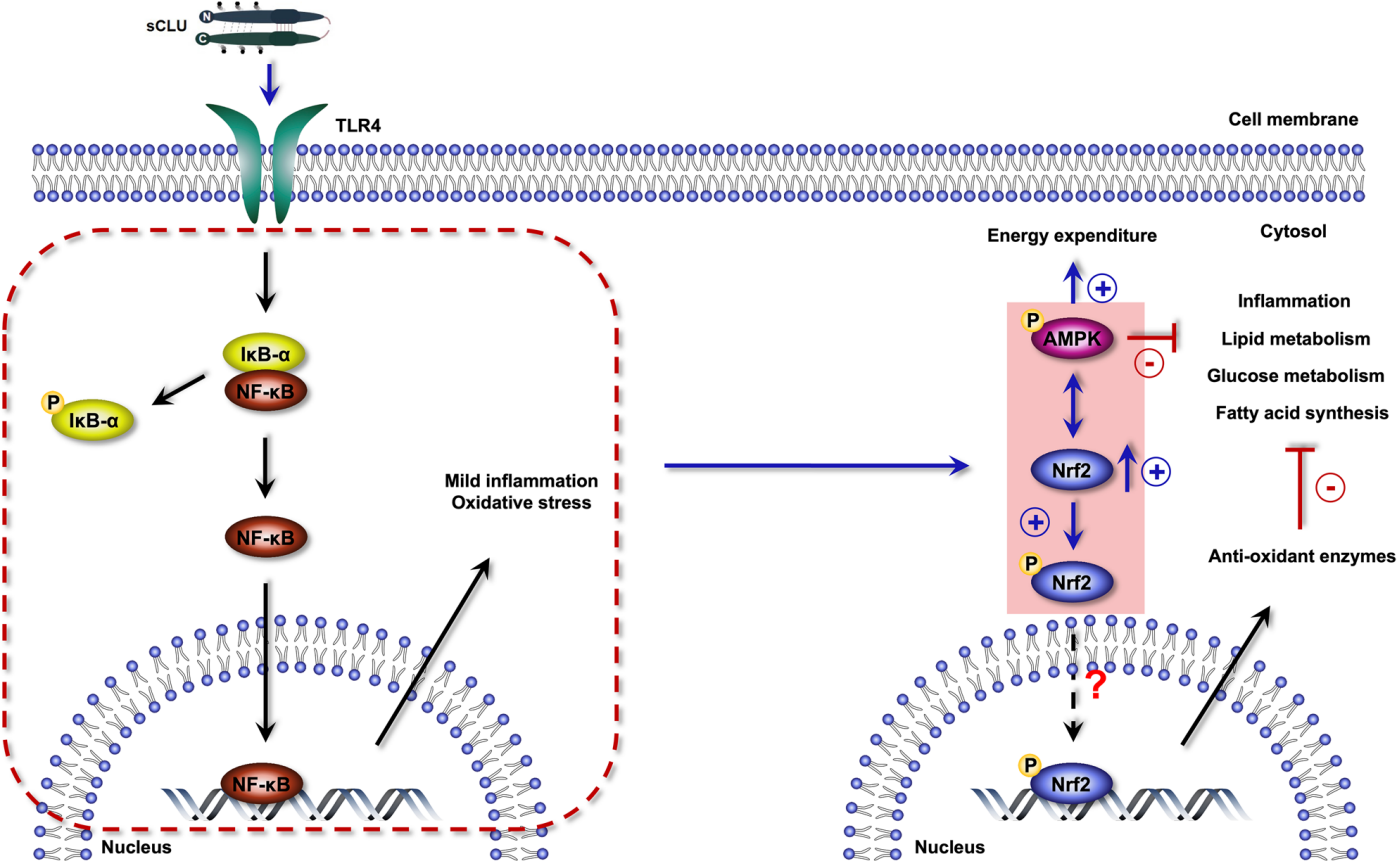
Schematic of the protective mechanism of clusterin via preconditioning. Clusterin induces a mild inflammatory response and oxidative stress, which enhances AMPK phosphorylation and Nrf2 expression, producing a preconditioning status.

Taken together, mild inflammation and oxidative stress caused by clusterin induce the activation of Nrf2 and AMPK, protecting against subsequent severe inflammation and oxidative stress (Fig. 6). Additionally, these results suggest that clusterin is a potential modulator of preconditioning, and that regulation of inflammation by clusterin may provide important clues for optimizing treatment strategies of various inflammation-related diseases.

## Methods

### Cell culture

Huh7 cell lines were purchased from the Korean Cell Line Bank (KCLB, Seoul, Korea) and were incubated in DMEM (Hyclone CO, Logan, UT, USA) supplemented with 10% fetal bovine serum (Hyclone CO, Logan, UT, USA) containing 1% antibiotics-antimycotics (Gibco-BRL, Rockvile, MD, USA) in a humidified CO_2_ incubator. Cells were sub-cultured and seeded into 6-well plates after reaching 70–80% confluence. Cells were cultured for 24 h, then cultured in serum-free DMEM for overnight. Huh7 cells were treated with purified human clusterin ^49^ with or without 1 h of preincubation with inhibitors.

### Fractionation of nuclear and cytosolic extracts

Nuclear and cytosolic extracts were prepared using the Nuclear/Cytosol Fractionation kit (Bio Vision, Milpitas, CA). The experimental procedure was followed by manufacturer’s instructions.

### Reagents and antibodies

TLR4 inhibitors (CLI-095 and OxPAPC) were purchased from Invivogen (San Diego, CA, USA). AMPK inhibitor (Compound C, CAS 866405-64-3) was purchased from Calbiochem (San Diego, CA, USA). Nrf2 inhibitor (ML385, CAS 846557-71-9) was purchased from Axon Medchem BV (Groningen, Netherlands). Antibodies for GAPDH (14C10), p-AMPK (Thr172, 40H9), AMPK (D13E1), and anti-rabbit IgG (HRP-linked, 7074) were purchased from Cell Signaling Technology (Danvers, MA, USA). Antibodies for clusterin (M-18, sc-6420) and Nrf-2 (C-20, sc-722) were purchased from Santa Cruz Biotechnology (Santa Cruz, CA, USA). Antibodies for α-SMA (ab32575), p-Nrf2 (ab76026), Lamin B1 (ab16048) were purchased from Abcam (Cambridge, MA, USA) and F4/80 (MCA497GA) antibody was purchased from Bio-rad (AbD Serotec, Oxford, UK).

### Animals and dietary experiments

wCLU-tg mice were generated by crossing floxed transgenic mice carrying clusterin knock-in allele (CLU-KI^tg+/+^)^28^ with transgenic mice carrying Cre recombinase under the control of the adenovirus EIIa promoter to drive Cre recombinase expression in the early mouse embryo (Jackson laboratory, stock number: 003724). Six-week-old male C57BL/6 wild-type and wCLU-tg mice (n = 8 to 10 per group) were used for experiments. Mice were independently fed WD (D12079B, Research Diet, NJ, USA) or CHOW (5L79, PMI Nutrition, North Arden Hills, MN, USA) for 15 weeks. Mice were housed with a 12:12 h light/dark cycle, with lights on at 8:00 and light-off at 20:00. All animal experiments were preformed according to the guidelines of the Korea University Institutional Animal Care and Use Committee (KOREA-2017-0197).

### Western blotting

Snap-frozen tissues were washed twice with PBS and dissolved on ice for 30 min to 1 h in RIPA buffer (50 mM Tris–HCl, pH 8.0; 150 mM NaCl; 1% NP-40; 0.5% sodium deoxycholate; 0.1% SDS) containing 50X protease inhibitor cocktail (Roche, Mannheim, Germany). Dissolved tissue and cell lysates were centrifuged (4 °C, 13,000 rpm, 10 min) and extracted proteins were quantified by Bradford assay, then boiled at 100 °C for 5 min. Quantified samples were subjected to SDS-PAGE (120 V, 1 h) using a 10–12% acrylamide gel. Proteins were then transferred (4 °C, 100 V, 1 h) to a nitrocellulose membrane (Millipore) that was then blocked with blocking solution (5% non-fat dry milk) for 30 min. After blocking, membranes were incubated at 4 °C for overnight with appropriate antibodies for the given experimental conditions. Next, membranes were incubated with HRP-conjugated secondary antibody at room temperature for 1 h. Enhanced chemiluminescence (ECL) solution was added to the membranes and protein bands were detected using X-ray film.

### Immunohistochemistry

Tissues were fixed in 10% Neutral buffered formalin (NBF) for 48 h, embedded in paraffin, and serial-sectioned at 4 μm thickness. Tissue sections were attached to microslides by incubation at 50 °C for overnight. Tissue sections were deparaffinized and rehydrated, then subjected to antigen retrieval at 100 °C for 30 min using 10 mM sodium citrate buffer (pH 6.0). Immunohistochemistry was performed by blocking tissue sections with normal serum at room temperature for 1 h, and then incubating at 4 °C for 12 h with the appropriate antibody. Tissue sections were then incubated with a biotin-conjugated secondary antibody at room temperature for 1 h, followed by incubation at room temperature for 1 h with an Avidin–Biotin Complex (ABC) solution (Vector Labs, Burlingame, CA, USA). Specific cells and proteins were stained using the 3,3′Diaminobenzidine (DAB) kit (Cell Signaling Technology, Danvers, MA, USA) and counterstained with Harris hematoxylin (American Master Tech Scientific, Lodi, CA, USA). The positive staining area was quantified NIH ImageJ software.

### Histology

The liver and fat sections were stained with Harris hematoxylin and eosin (H&E) for evaluation of histopathology and with Picro-Sirius red to assessment of hepatic fibrosis. The positive staining area was quantified NIH ImageJ software.

### Fat/lean mass measurement

The amounts of fat and lean mass were measured by an analytical instrument (Mini spec LF90II).

### Energy expenditure

Energy expenditure was measured using an indirect calorimeter. Mice were placed in a metabolic cage with water and food supplied. O_2_ and CO_2_ analyzers were calibrated to the gas standard, and oxygen consumption (VO_2_) and carbon dioxide emission (VCO_2_) were measured for 3 min, at 30-min intervals for 24 h.

### Statistical analysis

All data were expressed as standard error of the mean (SEM) and analyzed by ANOVA. Statistical analysis was performed using SPSS software and a p-value of less than 0.05 was considered statistically significant.

## Supplementary information

Supplementary Information.
