# Supplementary material for: Clusterin overexpression protects against western diet-induced obesity and NAFLD
Source: Sci Rep. 2020 Oct 15;10:17484. doi: 10.1038/s41598-020-73927-y (PMC7562726; doi:10.1038/s41598-020-73927-y)
Supplement: Supplementary file 1 — Supplementary Information. [file 41598_2020_73927_MOESM1_ESM.docx]

**Supplementary Information**

**Clusterin overexpression protects against western diet-induced obesity and NAFLD**

Jin-Sung Park^a,e1^, Woon-Kyu Lee^b^, Hyeon-Soo Kim^c^, Ji A Seo^d^, Dong-Hoon Kim^e^, Hee-Chul Han^a^, Bon-Hong Min^e^*

^a^ *Department of Physiology, College of Medicine, Korea University, Seoul, Korea*

^b^ *Department of Biomedical Sciences, College of Medicine, Inha University, Incheon, Korea*

^c^ *Department of Anatomy, College of Medicine, Korea University, Seoul, Korea*

^d^ *Department of Endocrinology and Metabolism, Korea University, Ansan, Korea*

^e^ *Department of Pharmacology, College of Medicine, Korea University, Seoul, Korea*

*Corresponding Author: [bhmin@korea.ac.kr](mailto:bhmin@korea.ac.kr)

**Supplemental Information**

**Supplementary Figure 1. Clusterin deficiency aggravates WD-induced metabolic diseases** (A, B) Weight gain and calorie intake between the two groups of mice fed Chow and WD for 15 weeks. Body weight and calorie intake of WD-fed CLU-KO mice were higher than that of WD-fed wild type mice. (C, D) Fat mass of WD-fed CLU-KO mice was higher than that of WD-fed wild type mice, but lean mass was not significantly different. (E) Oxygen consumption (VO_2_), CO_2_ emission (VCO_2_), and RQ value (respiratory quotient) were measured for 3 h at intervals of 30 min every 24 h. (F, G) H&E staining for eWAT and BAT of the two groups mice fed Chow and WD. **P* < 0.05; ***P* < 0.01; ****P* < 0.001 vs WD-fed WT mice. ns: not significant. Magnification, 200x, 400x.

**Supplementary Figure 2 Clusterin induces the expression of Nrf2 *in vitro*** Western blotting analysis of total Nrf2 expression. Huh7 cells were incubated in 6-well plate. After 24 hours, the cells were incubated in serum-free DMEM medium for 12 hours. Total Nrf2 expression in Huh7 cells was increased in a dose- (0, 0.1, 0.5, 1, 2, 4 µg/ml) dependent manner. GAPDH was used as an internal control

**Supplementary Figure 3. Correlation between TLR4 and clusterin/p-AMPK** Huh7 cells were pretreated with TLR4 inhibitors, CLI-095 (1, 5 µM), and OxPAPC (15, 30 µg/ml), for 30 min, then treated with clusterin (2 µg/ml) for 12 h.

**Original Figures (Western blotting, Figure 1,2, 5)**

**Figure 1**

**
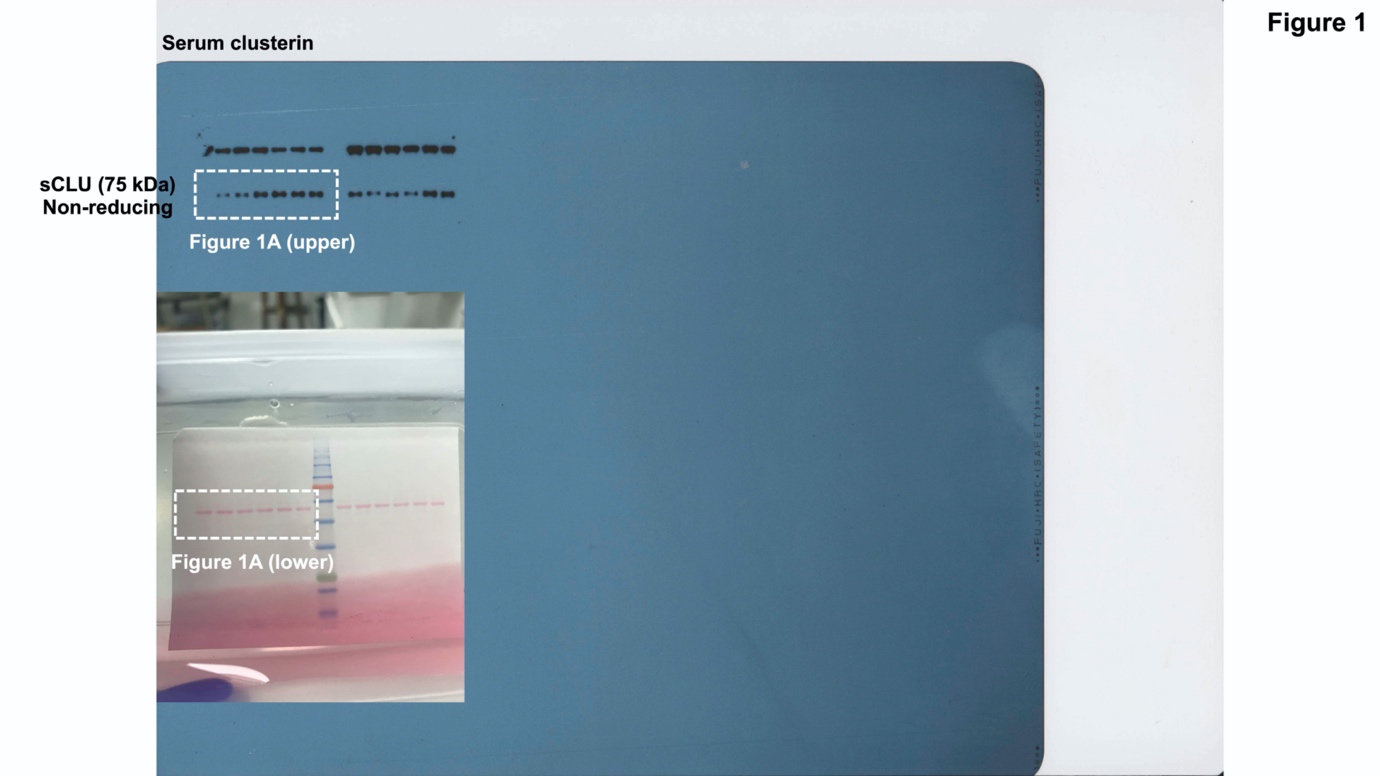
**

**
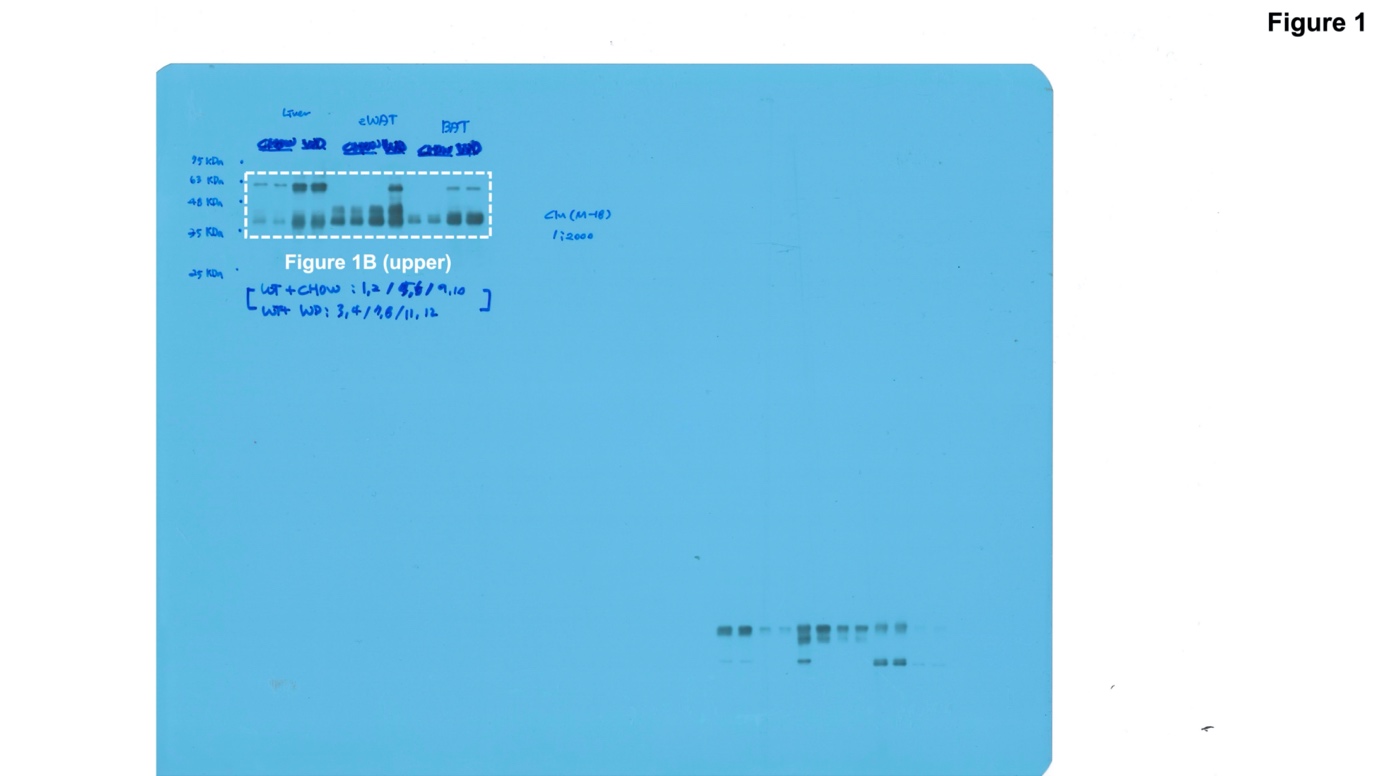
**

**
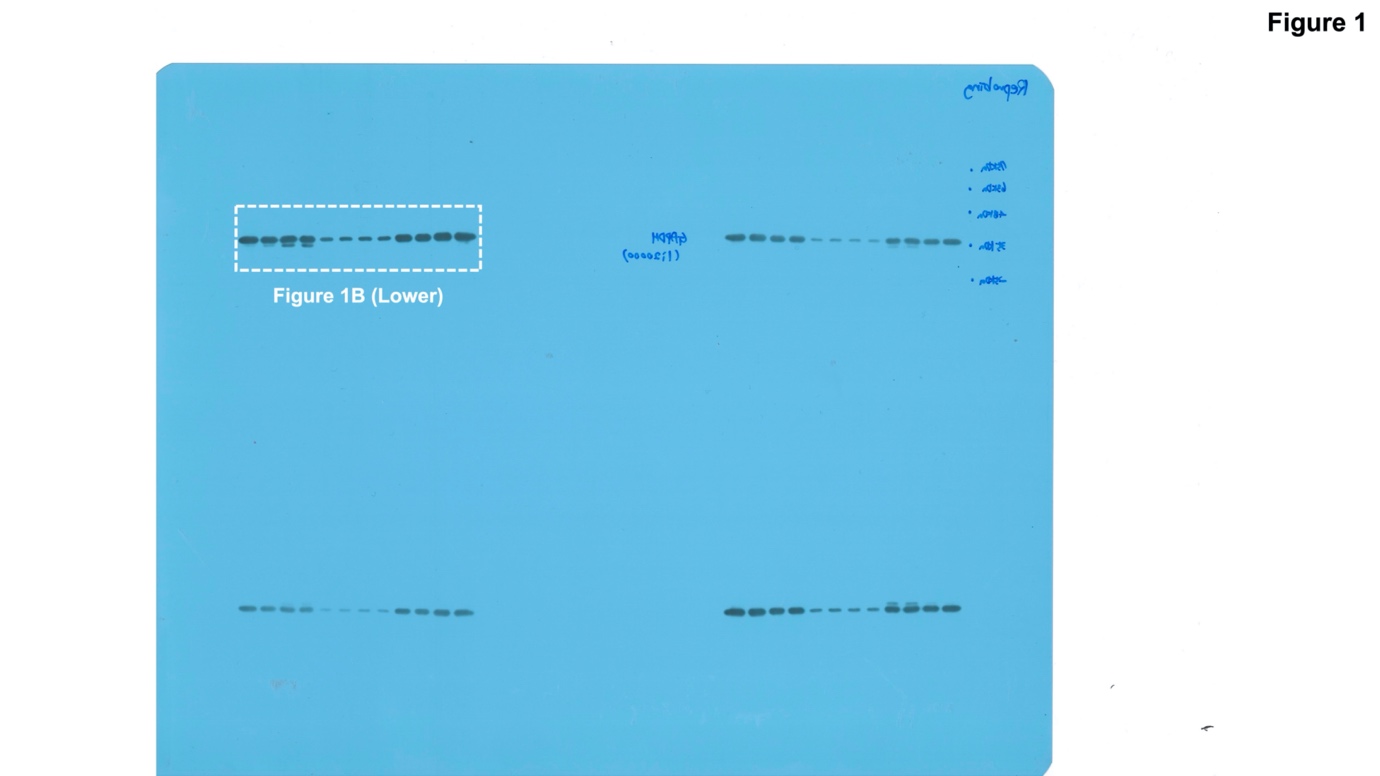
**

**Figure 2**

**A**

**
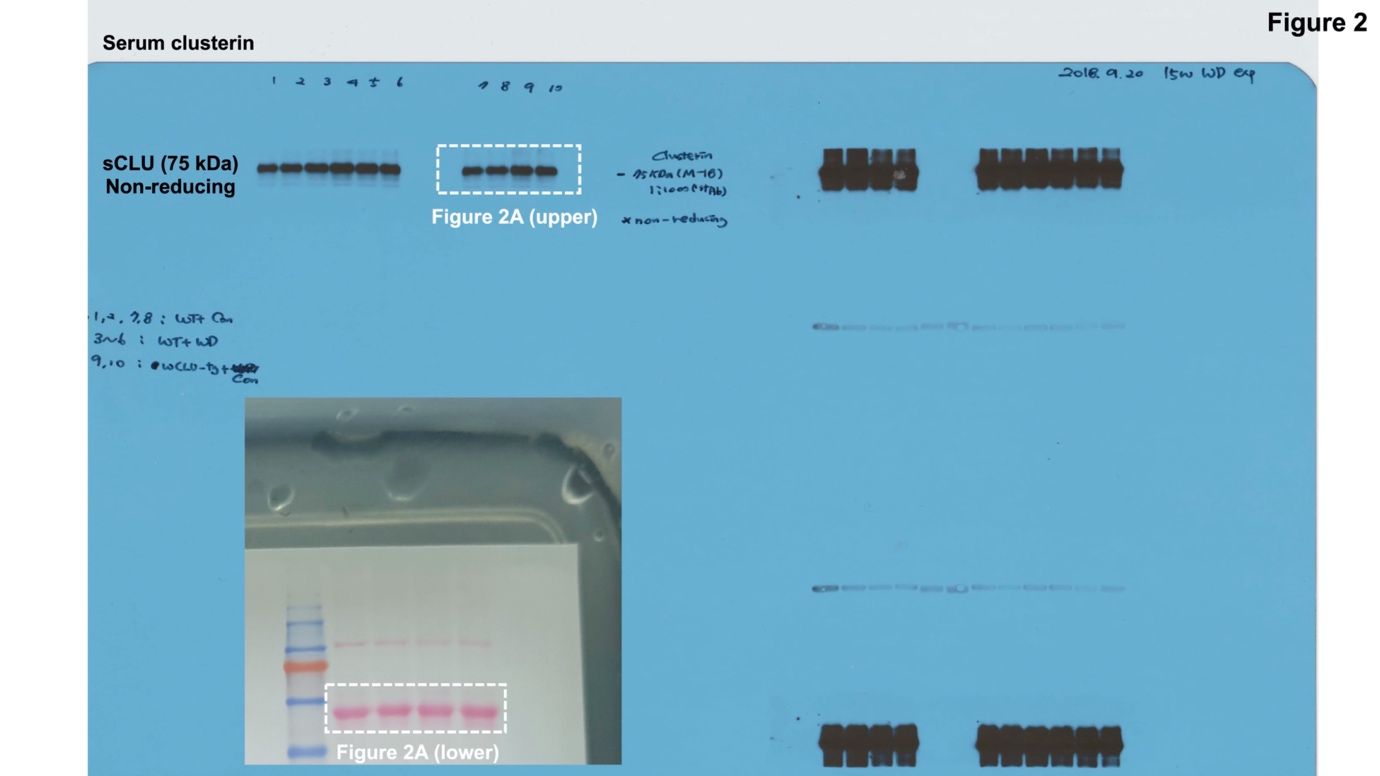
**

**B**


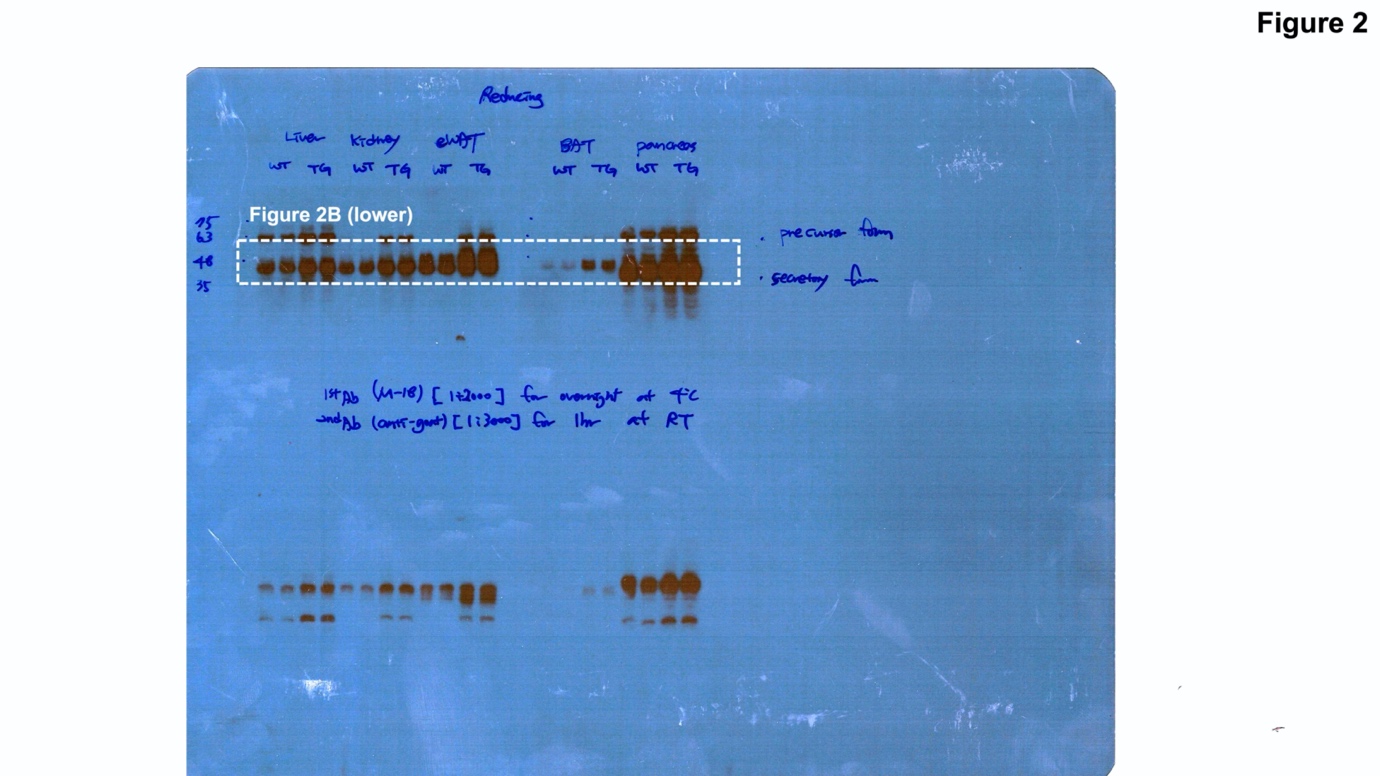


**
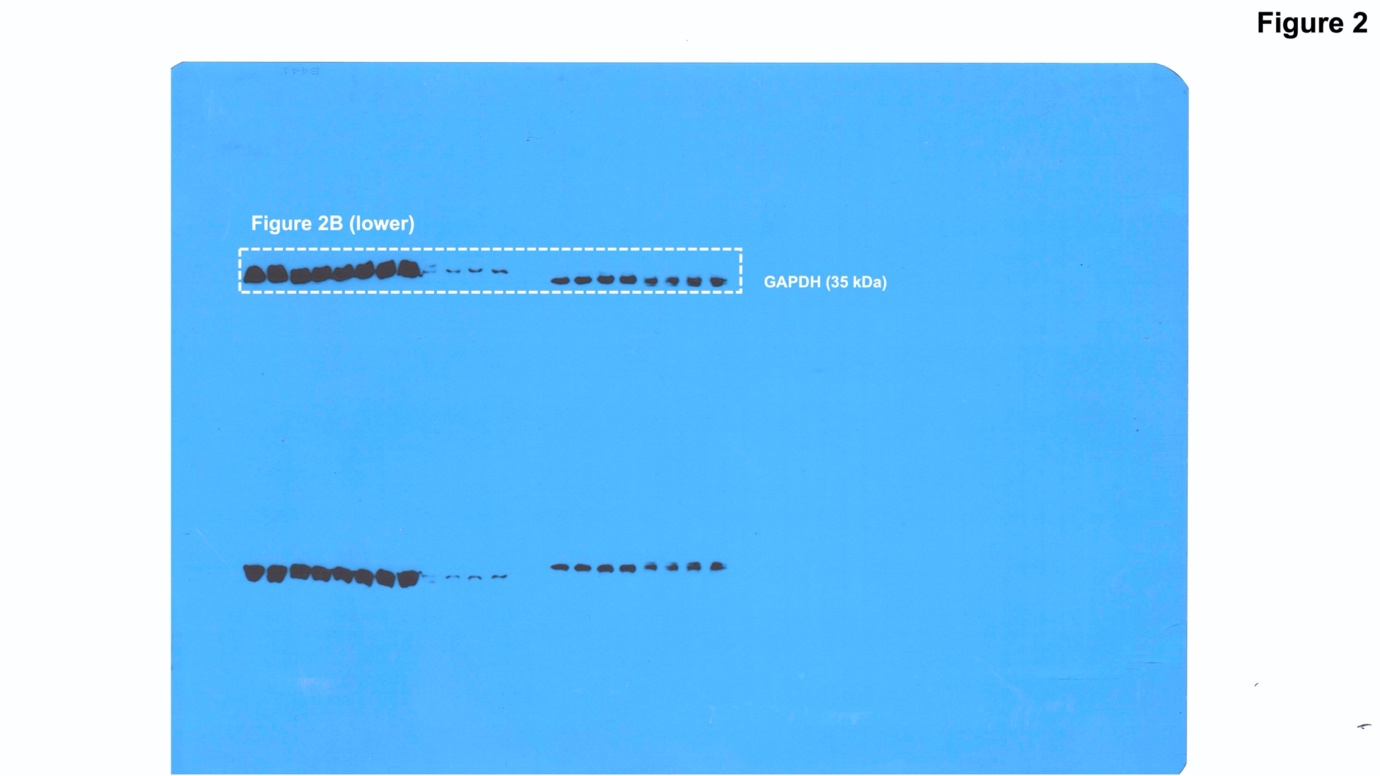
**

**Figure 5**

**A**

**
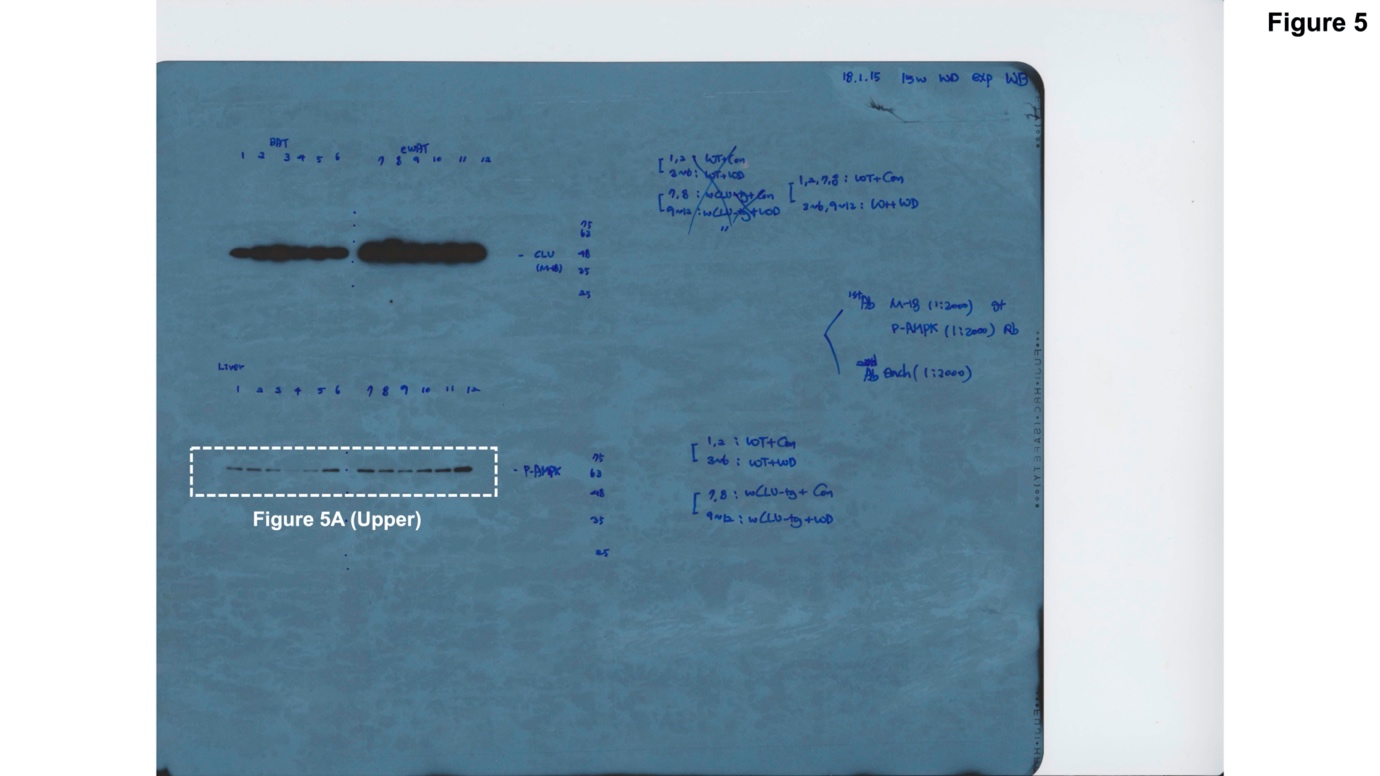
**

**
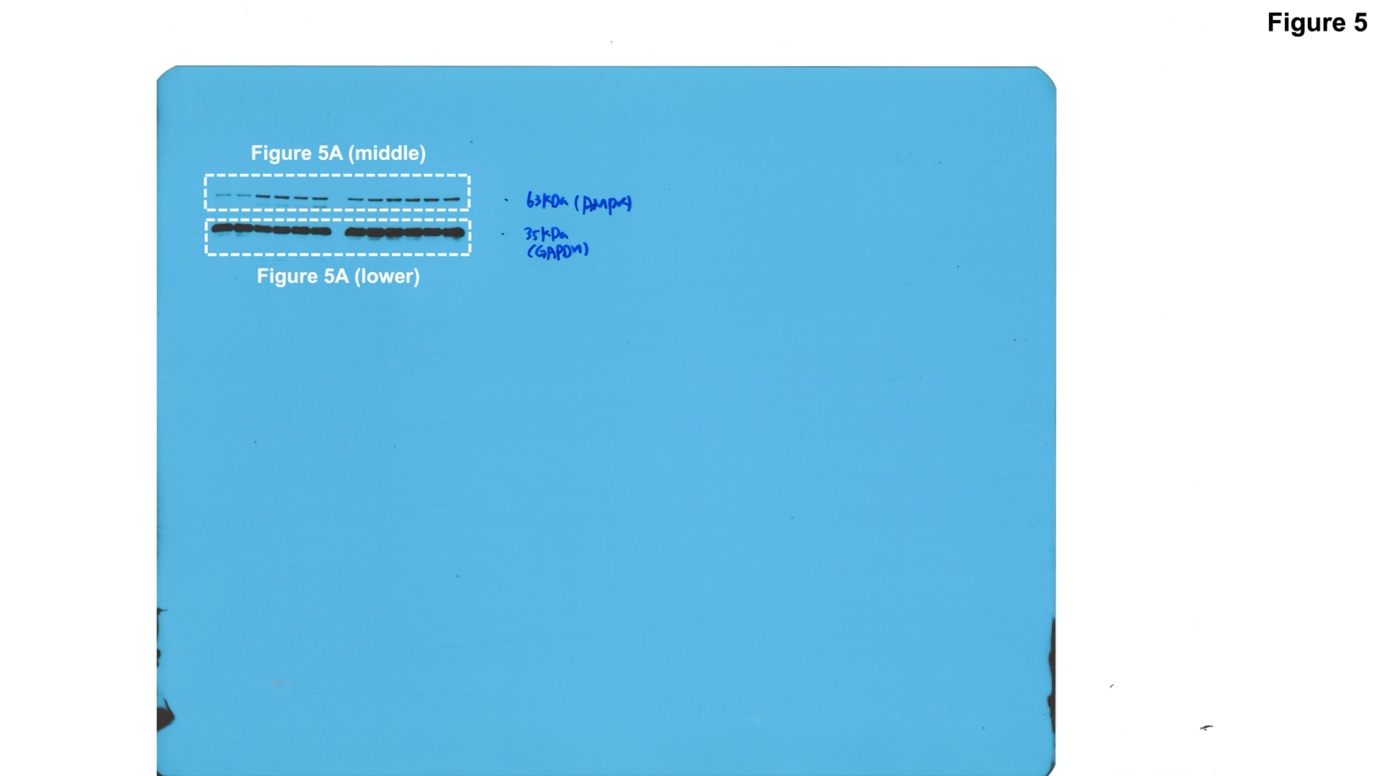
**

**B**

**
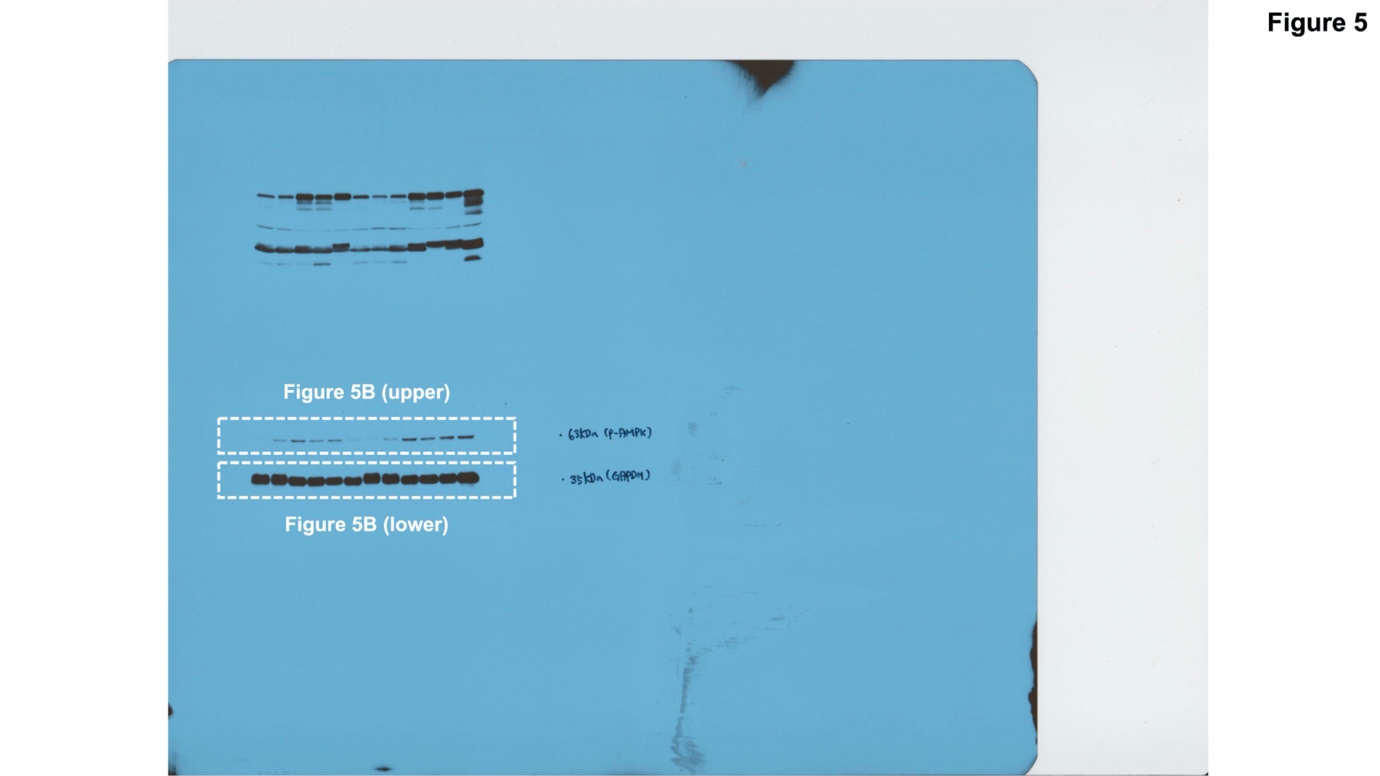
**

**
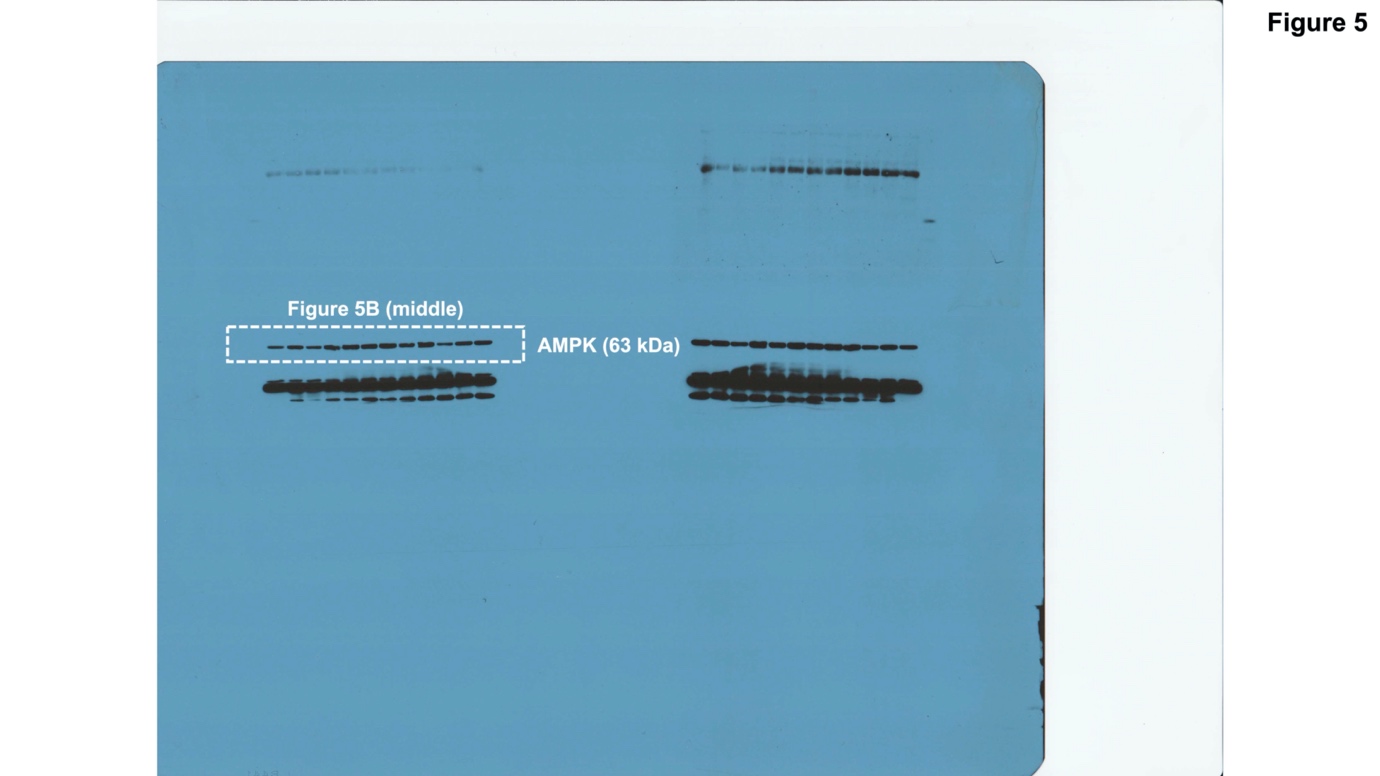
**

**C-D**

**
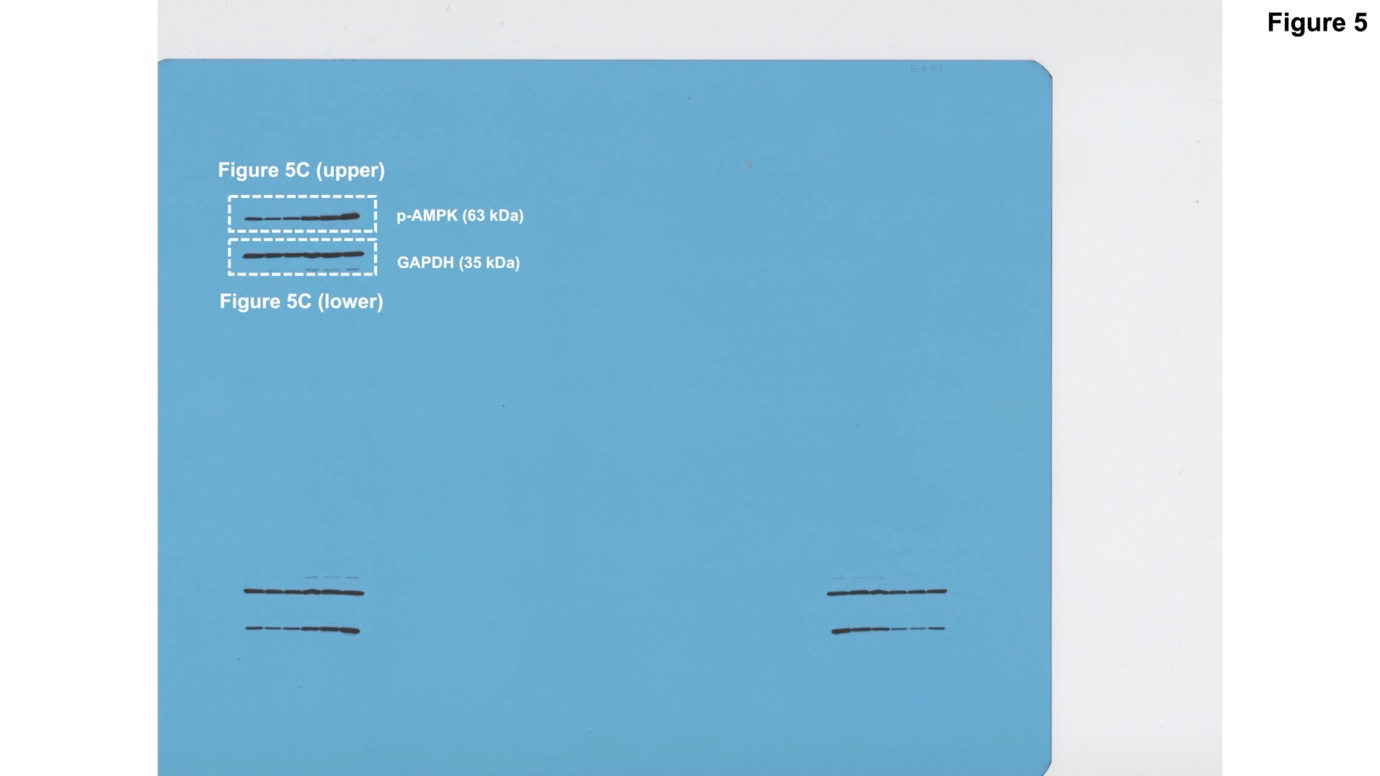
**

**
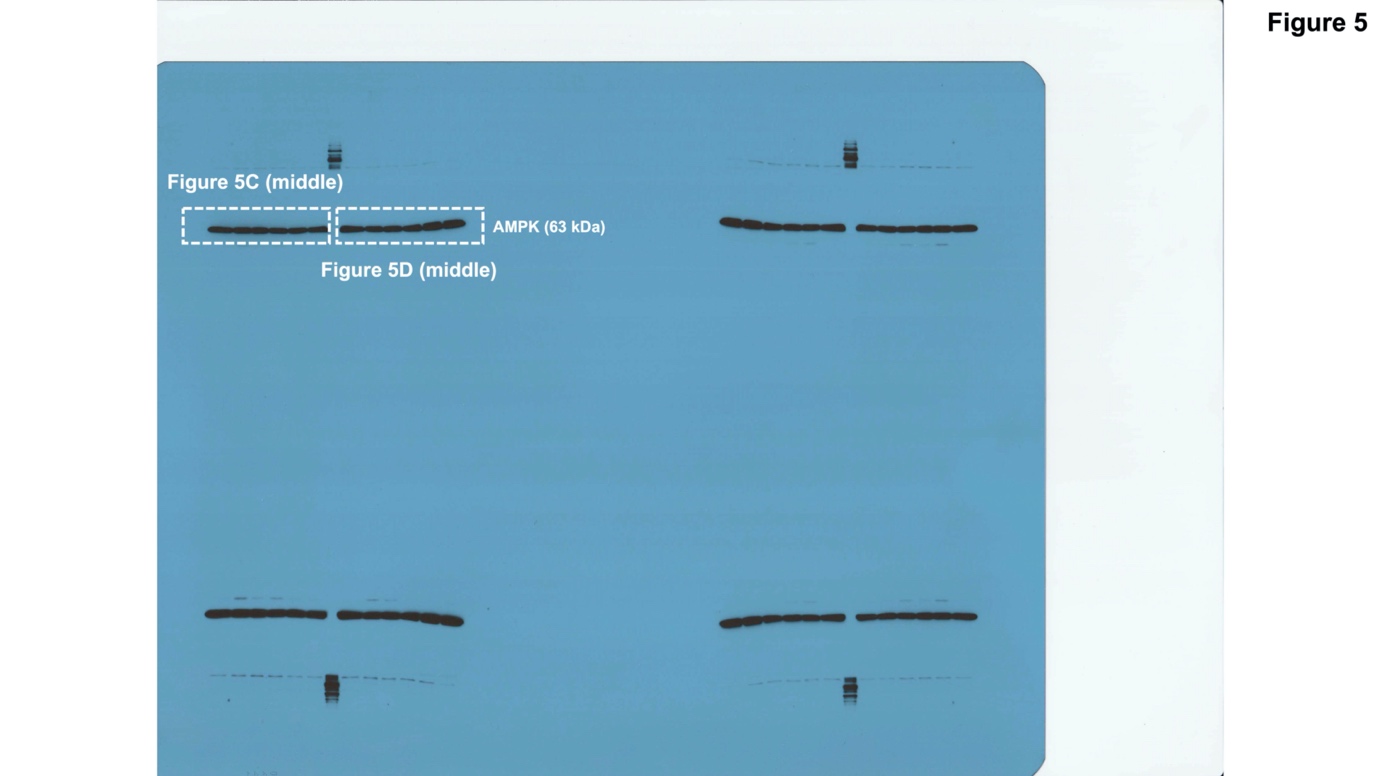
**

**
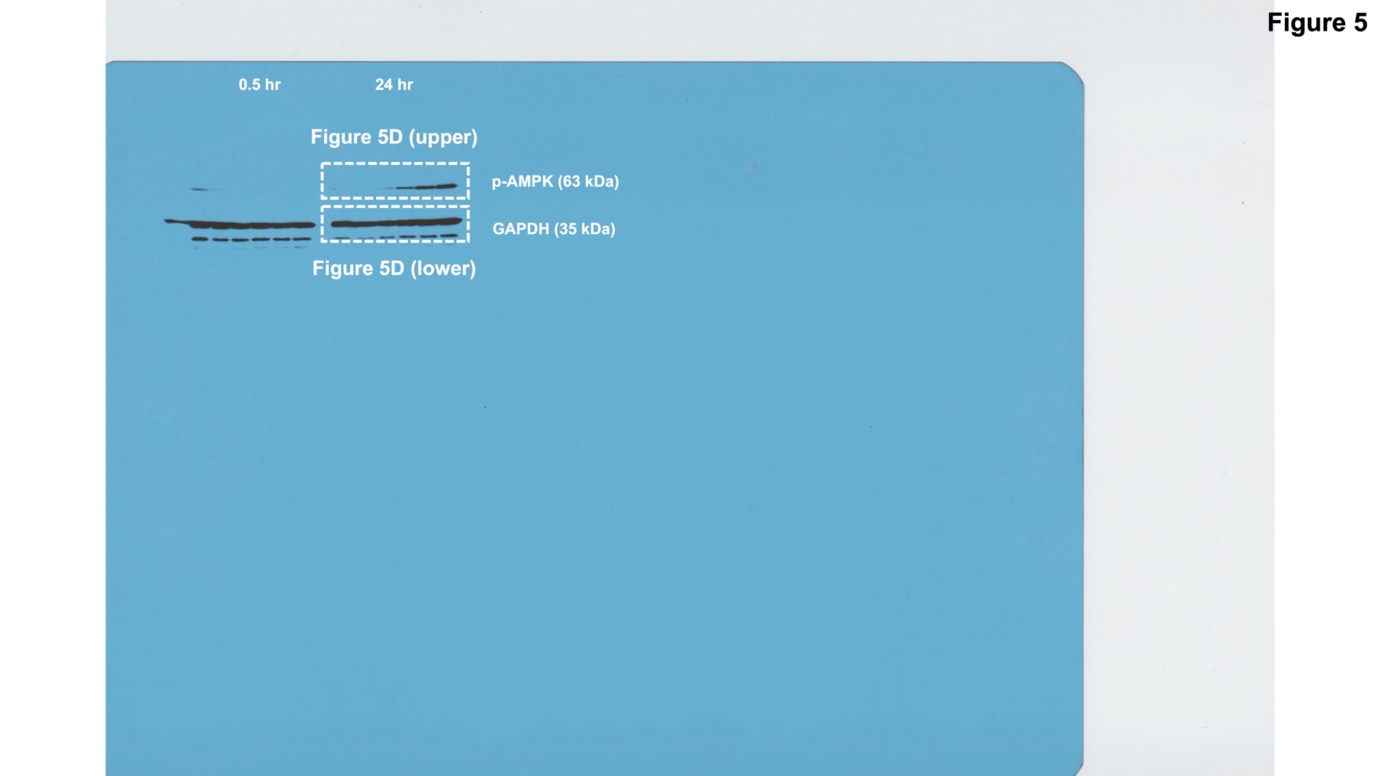
**

**E**

**
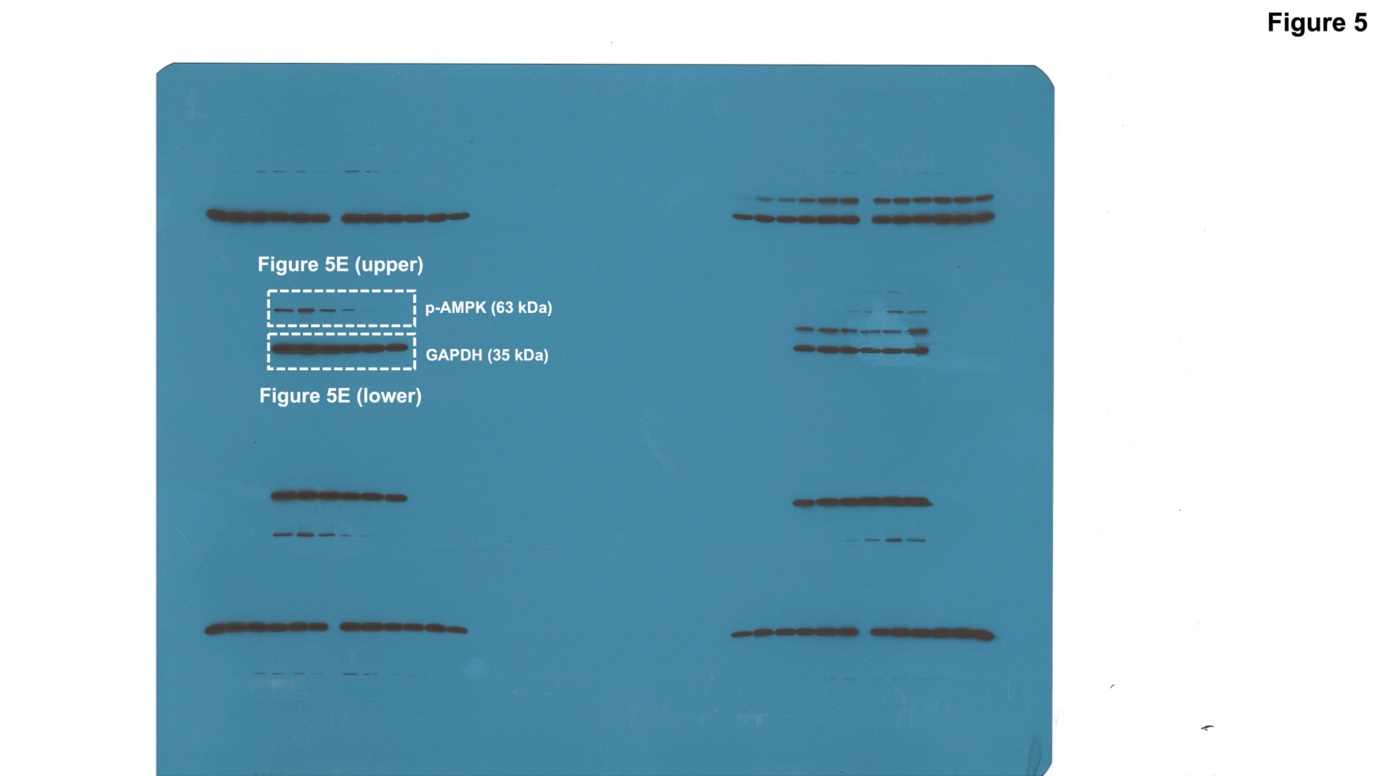
**

**
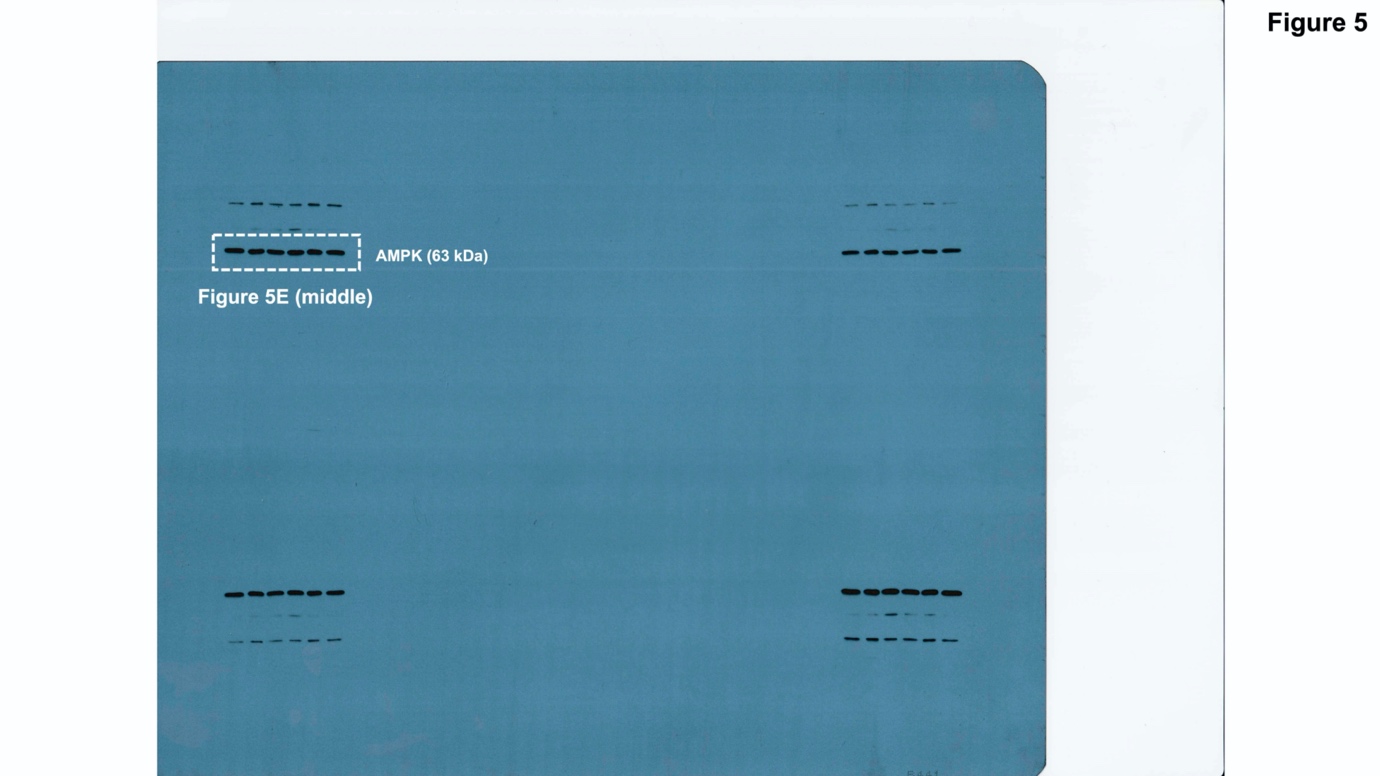
**

**F-G**

**
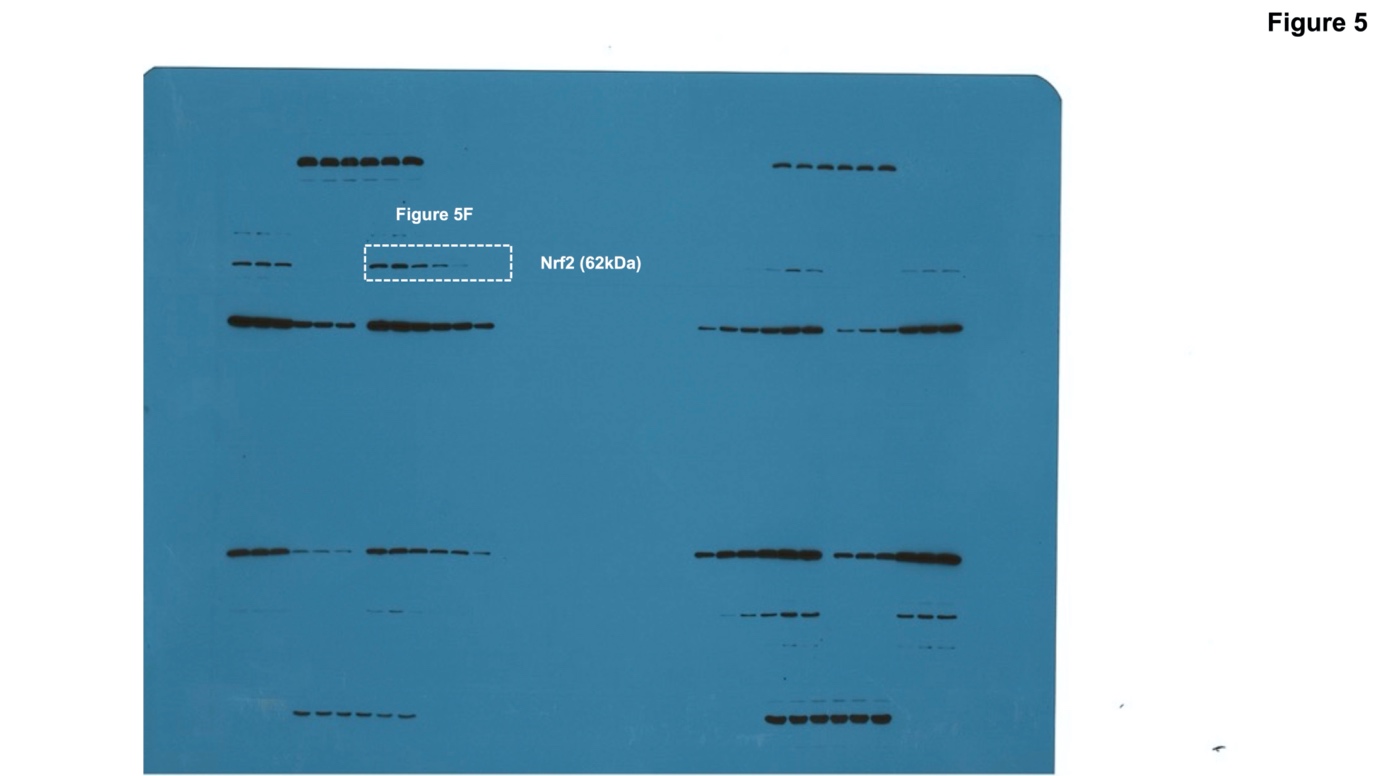
**

**
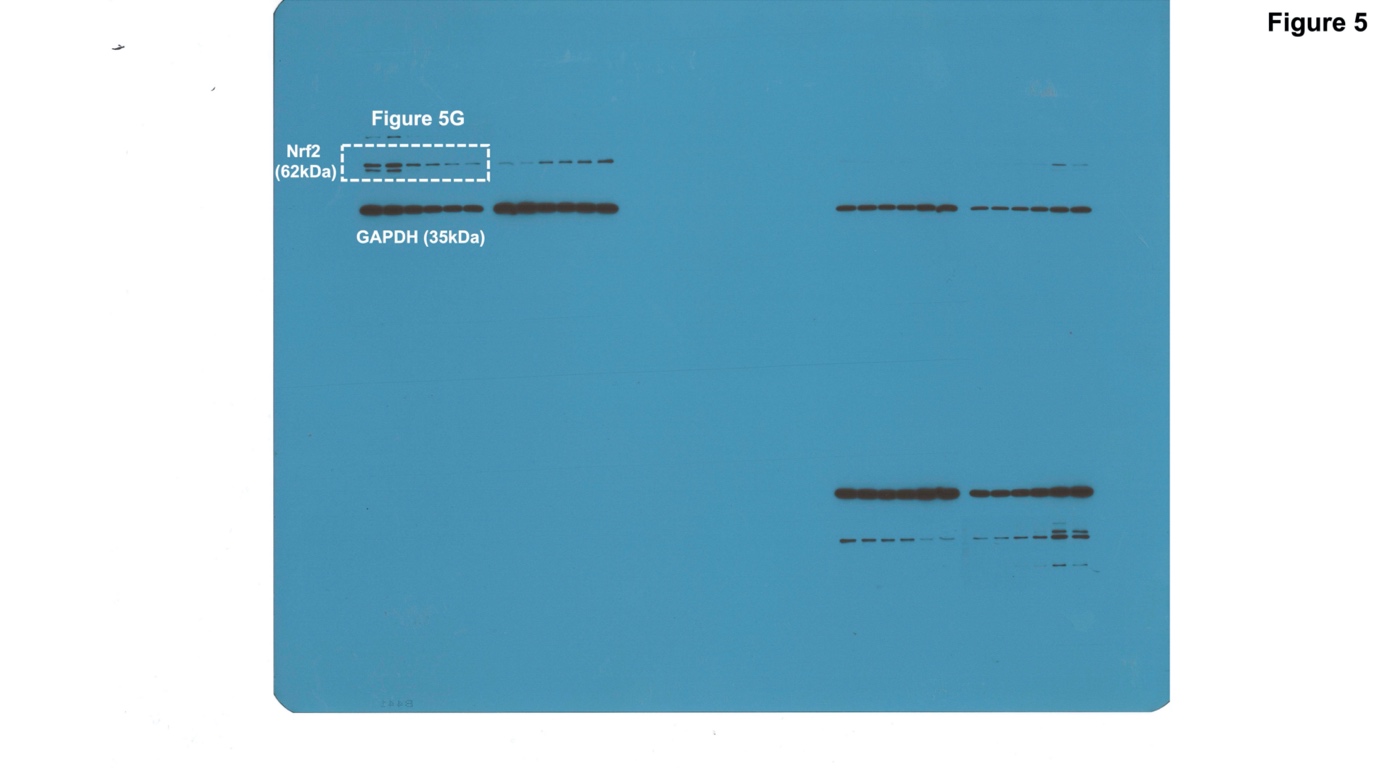
**

**
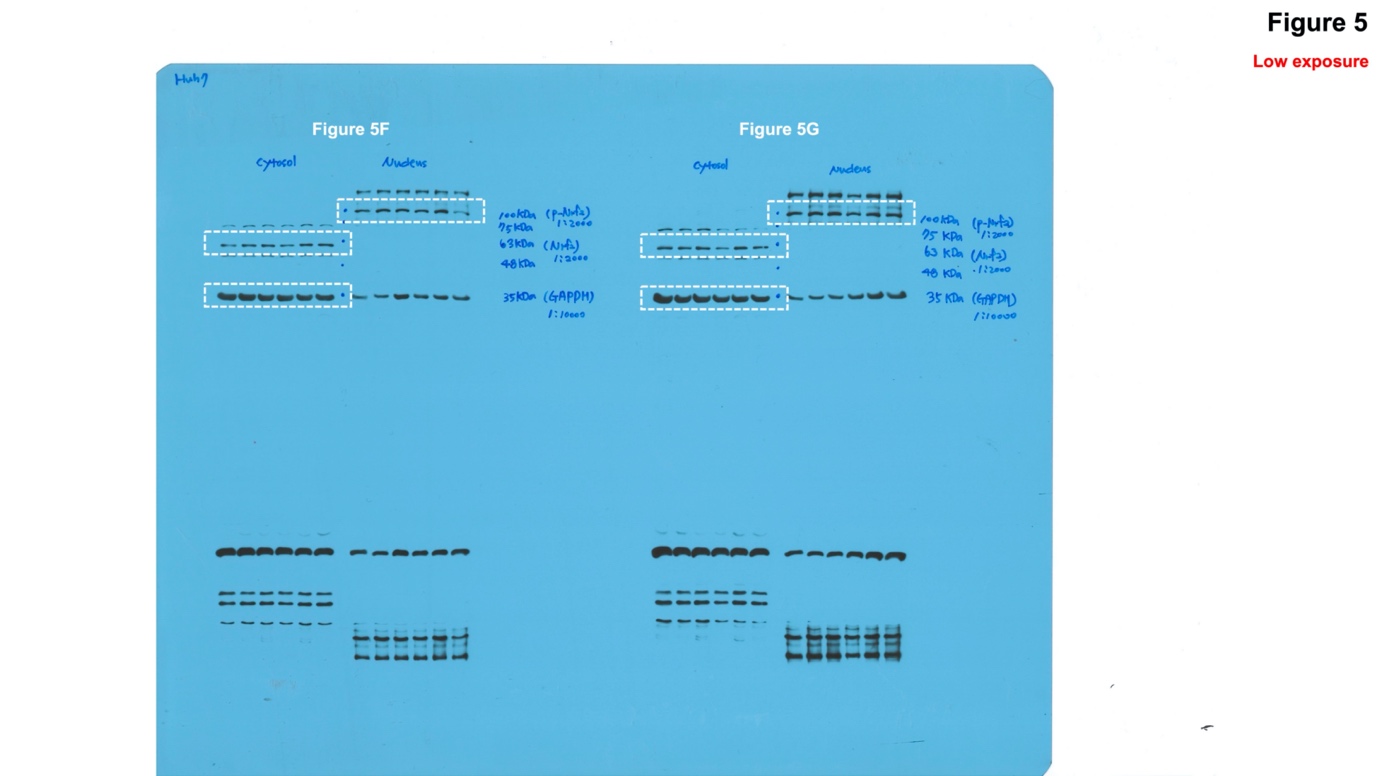
**

**
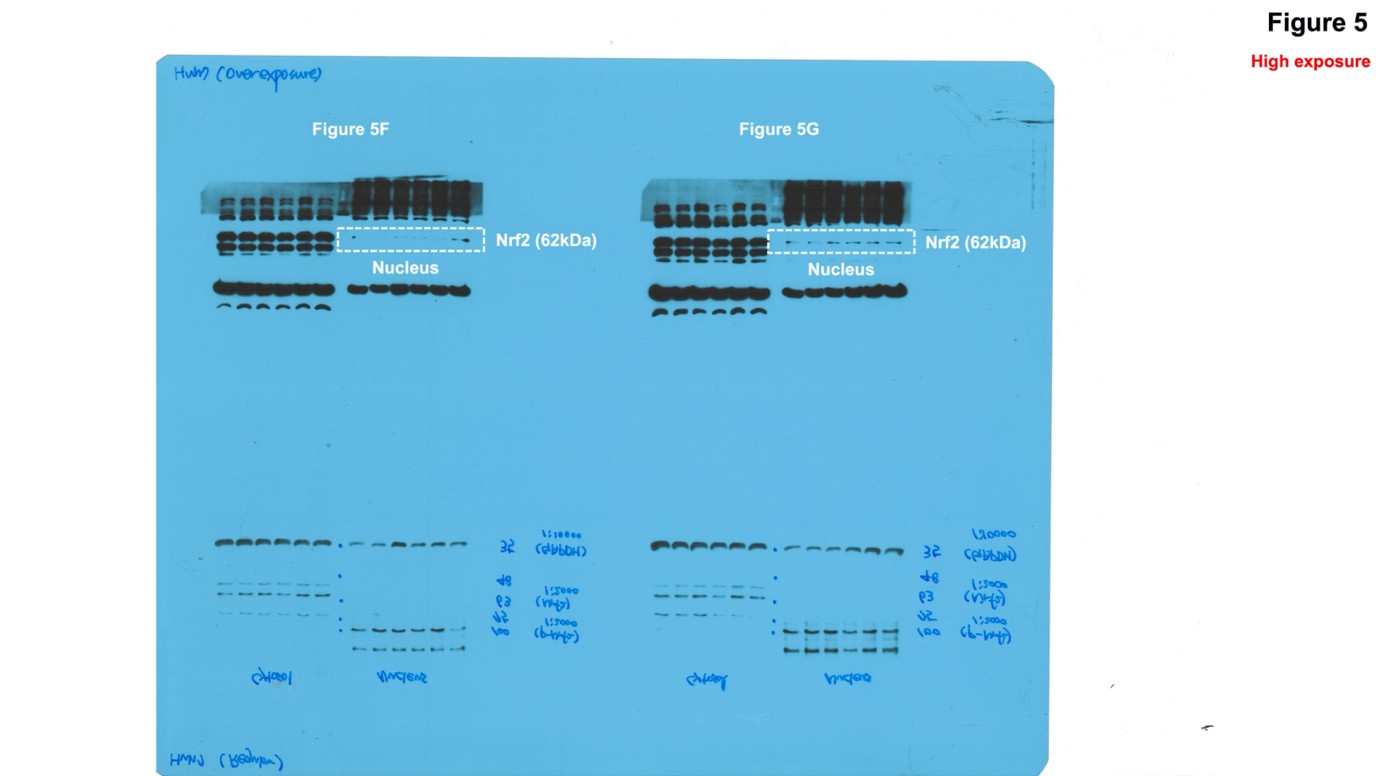
**

**
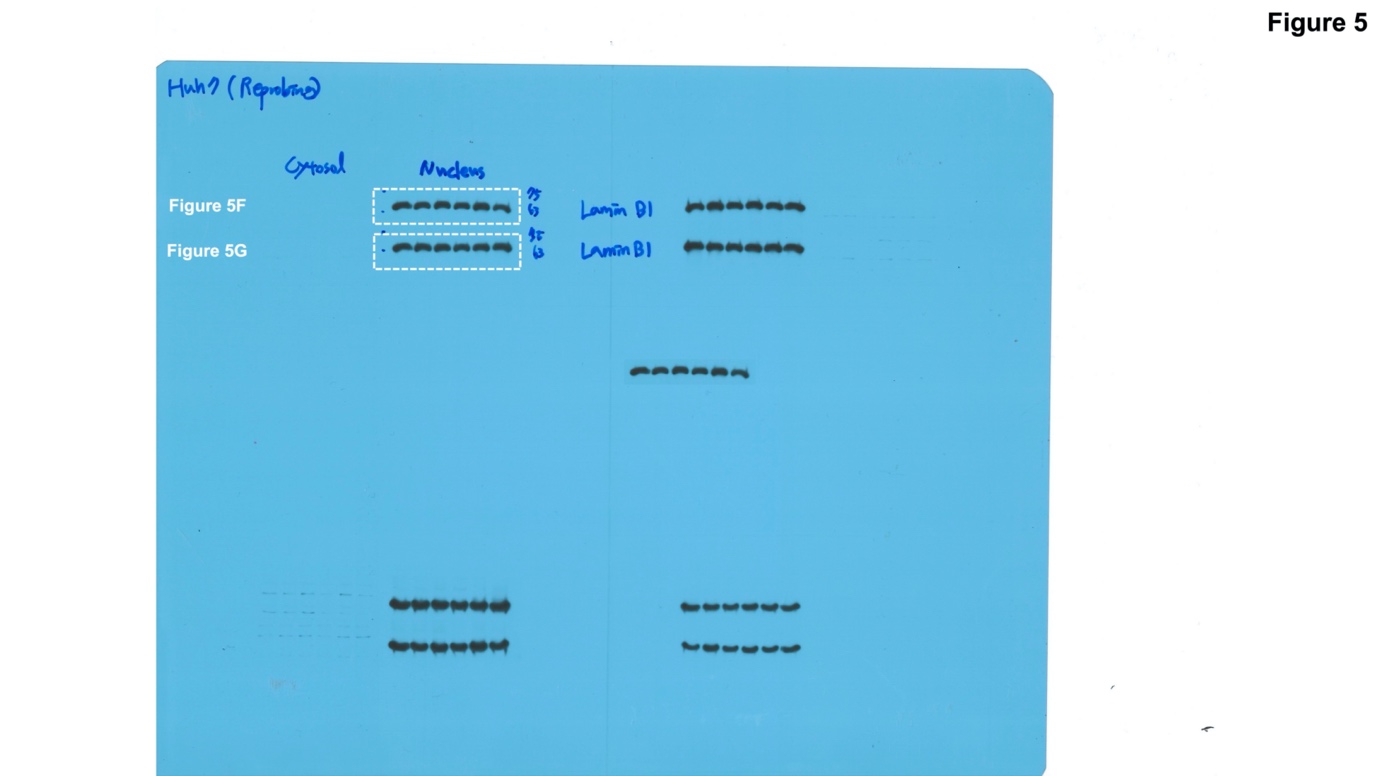
**

**
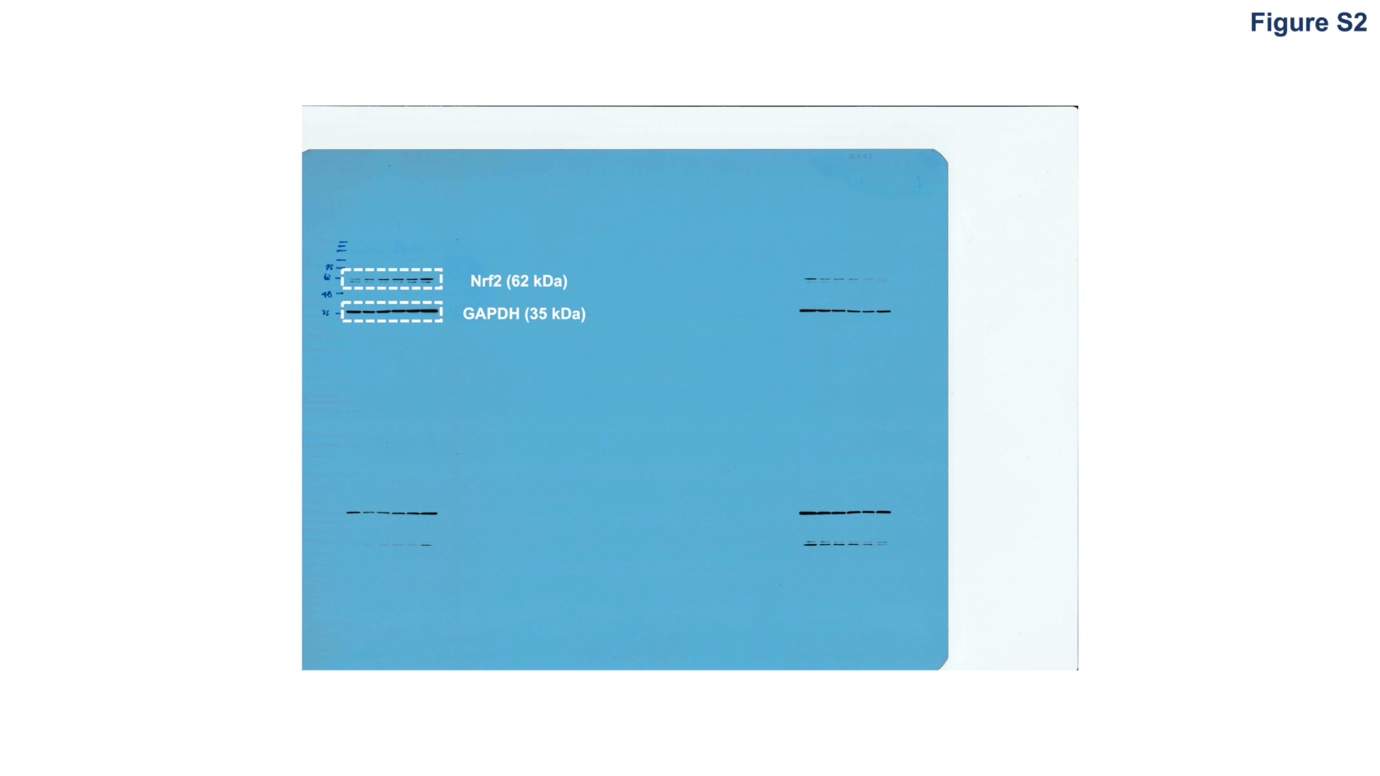
**

**Figure S3**

**
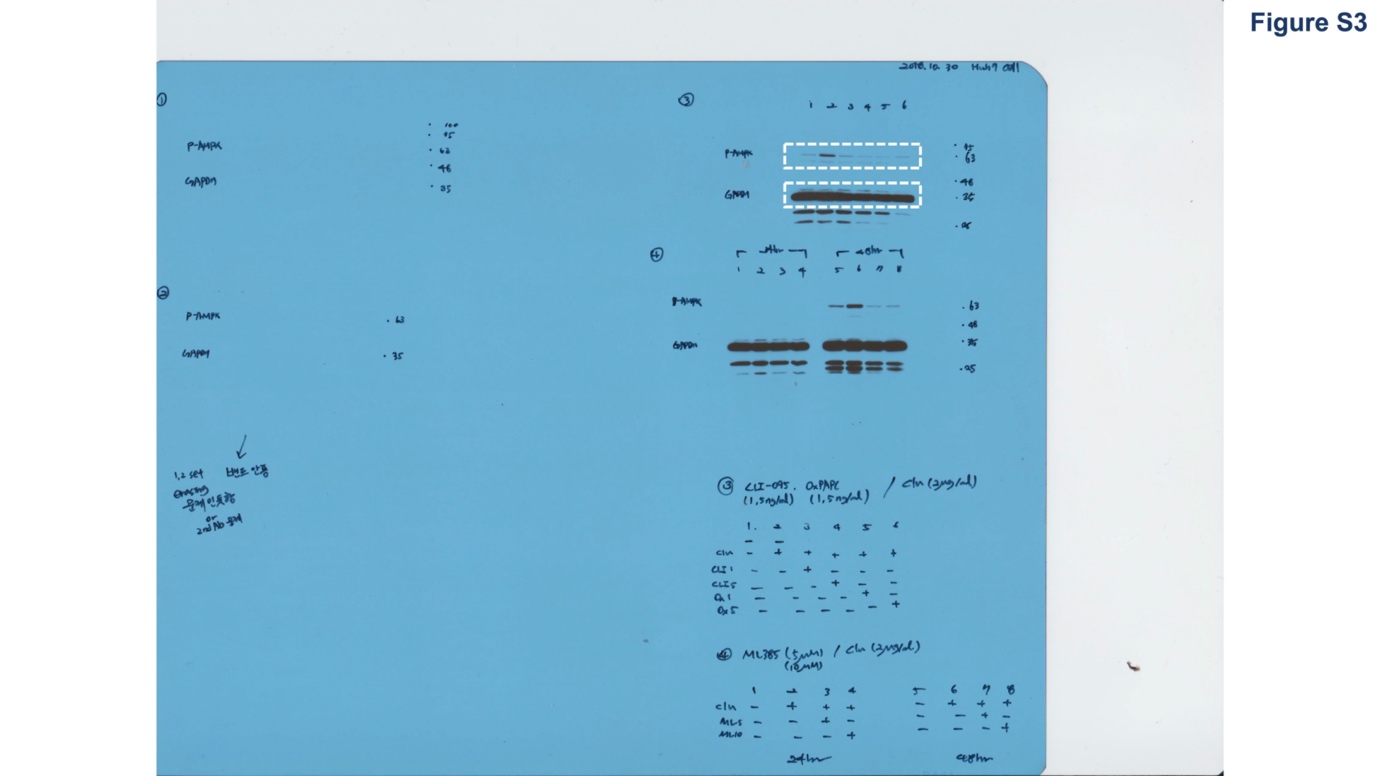
**
